# Integrative mindfulness-based infant parenting program: theoretical foundations and a novel intervention protocol

**DOI:** 10.3389/fpsyg.2025.1524008

**Published:** 2025-02-07

**Authors:** Or Burstein, Zipi Teshale Zevin, Ronny Geva

**Affiliations:** ^1^Department of Psychology, Bar Ilan University, Ramat Gan, Israel; ^2^The Gonda Multidisciplinary Brain Research Center, Bar Ilan University, Ramat Gan, Israel

**Keywords:** mindfulness, mindful parenting, infancy, group intervention, protocol, preterm birth, calming cycle, dialectical behavior therapy

## Abstract

Infancy is a formative period in which high-quality parental care plays a vital role in setting solid foundations that guide a child’s development. Mindfulness has been recognized for enhancing parental awareness and sensitivity to both self and child and can be utilized in clinical practice to facilitate healthy development. To adapt mindful parenting practice for implementation in pediatric care settings and the specific needs and challenges of parenting infants, the current study introduces a novel theoretical framework, combining mindfulness with elements from calming cycle theory, dialectical behavior therapy (DBT), trauma-informed care, emotion-focused therapy, schema therapy, and Vygotsky’s learning theory. These elements are not merely complementary but cardinal in meeting the diverse needs of parents during infancy, both in typical developmental contexts and following preterm birth, where additional stressors are often present. The study delineates the theoretical foundations of this integrative mindfulness-based approach and openly provides a novel comprehensive protocol of an 8-week group intervention program that operationalizes the proposed framework. This program focuses on enhancing parental mindful observation, non-judgmental acceptance, and goal-driven behavior to strengthen the resilience of the parent-infant relationship. Emphasizing the bi-directional nature of this relationship and the role of co-regulation with the child, the theory-derived program is designed to scaffold calming dyadic cycles, promote reconsolidation of birth-related adverse experiences, and facilitate flexibility in parental modes. The novelty of this intervention lies in its holistic approach to mindful parenting, conjoining diverse theoretical perspectives into a coherent, culturally adaptable, and clinically oriented protocol that can be assimilated in pediatric community clinics. The provided protocol may now enable the evaluation of the framework’s effectiveness in attaining positive effects for parents and children.

## 1 Introduction

The first 2 years of life represent a sensitive period for children’s emotional, social, cognitive, and neurological development (Curley and Champagne, 2016; Hodel, 2018; Nelson and Gabard-Durnam, 2020). While extensive research has focused on the negative impacts of early adversity (Doyle and Cicchetti, 2017; Liu et al., 2017), an equally important field explores pathways to resilience (Masten and Cicchetti, 2016). An essential factor in fostering resilience is the quality of care in the parent–child relationship (Darling Rasmussen et al., 2019), which can establish within the child a core sense of security that shapes how they interact and communicate with the world (Bowlby, 1988).

In early life, parents play a major role in nurturing children’s cognitive and emotional capabilities, influencing language acquisition (Madigan et al., 2019), emotion regulation (Groh et al., 2017), social skills (Pallini et al., 2014), cognitive performance (Han et al., 2023), and executive function development (Valcan et al., 2018). However, parenting during infancy years can be fraught with challenges, including heightened levels of fatigue (Badr and Zauszniewski, 2017), anxiety (Dennis et al., 2017), depression (Shorey et al., 2018), and posttraumatic stress symptoms (Yildiz et al., 2017). The risk of these issues is compounded for parents of infants with difficult temperaments, particularly following preterm births (Treyvaud, 2014; McQuillan and Bates, 2017; Helle et al., 2018).

Incorporating early-life parental interventions has been shown to promote resilience in typically developing populations and those who struggle with pediatric concerns (Puthussery et al., 2018; Jeong et al., 2021). Current parenting programs primarily focus on psychoeducation and facilitating parents’ ability to make meaning of and aptly respond to infants’ cues (Love et al., 2005; Nugent et al., 2007; Pontoppidan et al., 2016; Slade et al., 2020). Most of these interventions are grounded in attachment theory (Bowlby, 1988) and mentalization processes (Fonagy et al., 2018), emphasizing parental sensitivity and responsiveness as key factors in fostering the child’s secure attachment. Additionally, early interventions developed in neonatal intensive care units (NICUs) contexts aim to promote the bonding process by facilitating soothing physical and sensory communication (Welch et al., 2015; Ettenberger et al., 2021). These interventions leverage the neuroprotective effects of affective touch to strengthen parent–child co-regulation (La Rosa et al., 2024a). In recent years, the scope of early intervention has been broadened with mindfulness.

Mindfulness—characterized by intentional, non-reactive awareness of present experiences (Nhất Hạnh, 1987)—was first incorporated into parenting by Kabat-Zinn and Kabat-Zinn (1997). Meta-analyses revealed that mindful parenting group interventions effectively promote parental mindfulness (Shorey and Ng, 2021) and attenuate stress (Burgdorf et al., 2019). Most clinical studies have focused on parents of children above the age of 2 years, except for a relatively recent protocol (Potharst et al., 2017) that specifically targets parents of infants (i.e., 0–18 months) through an 8-session format including mother–child dyads emphasizing mindfulness exercises with the baby.

Despite recent progress, no existing mindful parenting intervention focuses exclusively on parents of infants (Kil et al., 2021). A parent-centered group intervention protocol allows for larger group participation, is applicable in pediatric community settings, enables the inclusion of both parents and enhances focus on parental needs. Moreover, we argue that traditional mindfulness skills alone may not fully meet the diverse needs of parents during this period. Addressing unresolved peripartum experiences and parents’ emotive-behavioral tendencies shaped by their own childhoods, along with developing additional skills like scaffolding effective learning and nurturing the tactile and emotional aspects of the relationship, are called upon. Thus, we propose an integrative mindful parenting model tailored to the specific challenges faced by parents in their child’s first 2 years, delineating the focal theoretical tenets and an applicative clinical intervention protocol.

## 2 Theoretical foundations

Here, we develop an infancy-oriented theoretical model of integrative mindful parenting, primarily grounded in mindfulness. We commence by reviewing existing models and noting gaps that our approach aims to address.

The pioneering work of Kabat-Zinn and Kabat-Zinn (1997, 2021) highlighted the importance of incorporating the core mindfulness elements of being present, compassionate, accepting, and emotionally aware into the parent–child relationship. Their model guides parents to listen attentively to their children’s verbal and nonverbal cues while remaining connected to their own emotions. They emphasized that parenting is inherently stressful and argued that mindfulness enables parents to respond more wisely rather than react automatically. They proposed that parenting transformation is construed through the lifelong cultivation of mindfulness and offered daily individual and interpersonal exercises to support this practice.

Duncan et al. (2009) built on the Kabat-Zinns’ philosophical underpinnings, adding a structured, research-based perspective, outlining key parental behaviors and attitudes that encapsulate mindful parenting, including listening with full attention, non-judgmental acceptance, emotional awareness, self-regulation, and compassion. This conceptual model formed the basis for a group intervention protocol (Coatsworth et al., 2014).

Bögels and Restifo (2014) developed a parallel protocol, later adapted for parents of infants (Potharst et al., 2017). Rooted in mindfulness, their approach focuses on reducing automatic parenting behaviors and cultivating mindful awareness of both self and child. Drawing from schema therapy (Young et al., 2003), it also encourages parents to explore and address their own parenting patterns and schemas shaped by their childhood experiences.

Both models focus on traditional mindfulness elements—mindful observation, acceptance, and compassion—through practices such as mindful breathing, body scanning, and mindfulness of daily routines. These approaches help parents engage attentively and compassionately with their children and modulate emotional responses during stressful moments. However, we argue that they overlook essential aspects crucial for promoting mindful parenting in infancy. These include explicitly focusing on the cyclical process of co-regulation between parent and child, behavioral strategies for managing distress, nurturing self-acceptance, trauma processing, deepening emotional engagement, and a Vygotskyan approach to facilitate learning. Our model explores the necessity and unique contributions of each of these components, beginning with core mindfulness practices and incorporating auxiliary elements to address specific parental needs that current models have not yet fully addressed.

### 2.1 Core—mindfulness and mindful parenting skills

Promoting parents’ mindfulness of their infants and their own needs is crucial for the child’s psychological development. By fostering greater receptivity to the child’s cues, mindfulness enhances parental sensitivity, reinforcing secure patterns of attachment-seeking behavior and supporting the child’s ability to regulate emotions (Zhang et al., 2019, 2022). We concur with existing mindful parenting models (Kabat-Zinn and Kabat-Zinn, 1997; Bögels and Restifo, 2014; Coatsworth et al., 2014), which emphasize the importance of parents’ ability to actively observe and describe current experiences—both their own and their child’s—in a non-reactive and accepting manner. It is important, though, to provide a clear description of the kernels of mindful practice.

Mindfulness could be distilled into five core principles: *observing* (focusing attention on the present; Lilja et al., 2013), *describing* (articulating experiences in descriptive language, without interpretations; Baer et al., 2006), *being present* (approaching life with openness and curiosity; Nhất Hạnh, 1987), *non-judgmentalism* (accepting the present as it is; Carson and Langer, 2006), and *goal-driven* (acting based on goals; Bögels and Restifo, 2014; Linehan, 1993).

Mindfulness skills can be categorized as global or interpersonal. While there is debate on whether to prioritize global- or interpersonal/parenting-related skills, we propose that both are complementary in enhancing mindful parenting. Global mindfulness fosters abilities such as focused attention and self-regulation (Schuman-Olivier et al., 2020), while interpersonal mindfulness extends these capacities into relational contexts, emphasizing non-reactive engagement with others (Pratscher et al., 2019). Research shows that proficiency in global mindfulness enhances interpersonal mindfulness, particularly in parenting (Kil et al., 2021, 2023; Parent et al., 2021). However, although traditional mindfulness-based stress reduction programs can mitigate parenting stress (Neece et al., 2024), they often fail to instill the specific skills needed for mindful parenting (Lunsky et al., 2017). Thus, nurturing both global and interpersonal mindfulness skills is essential. General mindfulness strengthens parents’ “mindful muscle” and builds foundations for mindful engagement, while interpersonal mindfulness calibrates these skills to parenting practices (Parent et al., 2016).

To ensure accessibility for diverse populations—especially those skeptical or antagonistic toward mindfulness (Sobczak and West, 2013)—adaptations are required. Unlike previous authors (Nhất Hạnh, 1987; Kabat-Zinn, 1994) who endorsed embracing mindfulness as a more total and spiritual way of being, we advocate a more inclusive and mundane approach in which each individual observes and learns for themselves how mindfulness could be practical for them (Foale et al., 2024). Consequently, mindfulness could be seen as a tool that can aid us in everyday life, encouraging parents to integrate it in ways that fit their beliefs and serve their goals.

Studies demonstrated that non-judgmental acceptance is a pivotal mindfulness domain for promoting effective parenting and better child outcomes (Geurtzen et al., 2015; Corthorn and Milicic, 2016; Moreira and Canavarro, 2018). Early parenthood often brings feelings of guilt, shame, and self-judgment (Law et al., 2021), especially following preterm births (Ionio et al., 2016; Gonçalves et al., 2020). These feelings can negatively impact parental well-being, functioning, and bonding processes with the child, which might, in turn, hinder the development of a secure attachment of the child to the parent (Caldwell et al., 2021). Thus, non-judgmentalism should be regarded as one of the most valuable drivers of mindful parenting during infancy.

When conceiving a mindful parenting model for infancy, mindfulness practices should align with the unique challenges that parents encounter during this stage. Pressing concerns like sleep deprivation, physical demands, and emotional depletion (Martins, 2019) must be acknowledged. Furthermore, infancy-oriented mindfulness should emphasize attention to the infant’s nonverbal behavior and enhance awareness of the parent’s emotional and physical state. This approach ensures mindfulness is relevant to the realities of early parenthood, calling for a developmental model that centers on the main themes of parenting infants and the associated demands of self- and co-regulation.

### 2.2 Calming together

Linehan’s biosocial theory posits that emotion regulation deficits arise from the interaction between a child’s psychobiological predisposition and their environment’s responsiveness (Crowell et al., 2009). Rather than focusing separately on the child or parent, the theory concentrates on the goodness of fit between them (Chess and Thomas, 1991). By accounting for both the child’s traits and the parent’s propensities, it offers a conceptual compass for fathoming and navigating through the bi-directional nature of the parent–child relationship. It suggests that effective parenting entails attending to the child’s needs while acknowledging the mutual influence between parent and child. This is particularly relevant in infancy, where parental care can proliferate through awareness of child-instigated effects (Frosch et al., 2021).

The calming cycle theory extends these notions, suggesting that parent and child co-regulate each other. Through sensory contact and emotional communication, they often discover their ability to calm one another, reinforcing their “calming reflex” and fostering bonding experiences (Welch, 2016). Frequent disruptions to this process can result in adverse conditioning. Rather than supporting proximity-seeking behaviors, reflexes of avoidance may develop, leading to dysregulation in the autonomic states of both parent and child and conditioning them toward disconnection during physical contact. However, repeated calming interactions can restore the calming reflex and reestablish effective co-regulation of autonomic states, fostering a healthier parent–child relationship. This process highlights how bonding behaviors lay the foundation for the child’s development of a secure attachment to their parent (Ettenberger et al., 2021) while emphasizing its bi-directional nature.

The focus on repeated, tactile-affective loops, complements broader research on bio-behavioral parent–child co-regulation (Tronick, 2007; Wass et al., 2019; McGowan and Delafield-Butt, 2022). While many theoretical models describe the intricate process of mutual regulation in early interactions, calming cycle theory offers a straightforward, practice-focused approach that aligns well with mindful parenting—specifically by accentuating how soothing tactile contact can mitigate distress and deepen parent-infant bonding. Indeed, the calming cycle’s clinical approach encourages parents, even when distressed, to stay physically and emotionally engaged with their baby, expressing and communicating their emotions (Welch, 1988). This approach has been shown to improve children’s autonomic regulation (Porges et al., 2019; Lipschits and Geva, 2024), as well as cognitive, emotional, and social outcomes (Welch et al., 2015; Beebe et al., 2018). While the clinical implementation of this approach has primarily been studied in NICU settings, its principles can be applied in broader contexts to support all infants throughout their first 2 years of life.

We suggest that mindfulness forms the foundation of the calming cycle approach, and therefore nurturing it within a mindfulness-based agenda is highly fertile. Mindfulness encourages parents to *observe* and *describe* their emotions while *being present* and *non-judgmental*. By developing skills of mindful emotional expression, parents are more likely to maintain soothing tactile contact with their child, even during challenging moments. The calming cycle approach could be further enriched by introducing dialectical behavior therapy (DBT) strategies that help parents validate their emotions and effectively manage distress.

### 2.3 Evolving dialectically between acceptance and change

DBT—an evidence-based treatment for severe emotion regulation deficits, including self-harm (Kothgassner et al., 2021) and externalizing behavior (Jakubovic and Drabick, 2023)—incorporates mindfulness as a core component (Linehan, 1993). Beyond mindfulness, DBT provides valuable elements, including validation and distress tolerance strategies, which can further enhance the mindful parenting framework.

Validation is the act of communicating to others (or ourselves) that their (or our) responses, feelings, and thoughts make sense and are meaningful within a specific context (Linehan, 1993, 2015). Key assumptions include that individuals are doing their best, problem behaviors have underlying reasons, and recognizing them can open prospects for more constructive solutions. In the context of parenting, validation plays a pivotal role in acknowledging the emotional and physical challenges parents face, fostering self-compassion, and reducing feelings of shame and guilt. By validating their own experience, parents can remain more engaged with themselves and their child (Smith et al., 2023). Studies indicate that validation can reduce adverse emotional and behavioral manifestations, such as adolescent self-harm (Adrian et al., 2018), emotion dysregulation (Shenk and Fruzzetti, 2014), and negative affect in adults (Benitez et al., 2019, 2022). Assimilating validation as a primary skill is particularly crucial during infancy, a period marked by increased parental stress and a need for sensitive and responsive caregiving.

Problem behaviors often arise during heightened distress. A core aspect of DBT is distress tolerance, with skills oriented at either altering painful experiences or accepting them when change is not viable (Linehan, 1993). Such skills are vital as deficiencies in managing distress can lead to harmful parental behaviors (Rodriguez et al., 2017; Hajal and Paley, 2020). In parenting, the concept of distress tolerance extends beyond the individual level, as suggested in DBT, to encompass interpersonal distress tolerance, where parents regulate their own distress while simultaneously pacifying their child’s arousal. Mindfulness is essential, as it enables parents to identify and acknowledge painful experiences—a critical first step toward effective regulation. This approach is highly relevant in infancy, where exhausted parents require actionable strategies to address acute distress while with their child. Traditional mindful parenting models often lack these active strategies for behavioral change. Another sturdy factor that might thwart the attenuation of parental distress is the presence of unresolved adverse experiences from the early stages of the baby’s life.

### 2.4 Reconsolidating stressful experiences in a reassuring milieu

Maternal birth-related posttraumatic symptoms are estimated at 6.7% in typical circumstances and up to 21.1% in targeted samples, such as mothers who experienced preterm delivery (Heyne et al., 2022). Moreover, approximately 33–45% of mothers perceive their birth experience as traumatic (Beck et al., 2013). When discarded, segregated, or dissociated, trauma can cause severe distress, affecting sleep, emotion regulation, behavior, and social functioning (Herman, 1992; Geva et al., 2005; Yehuda et al., 2015). Parental posttraumatic stress disorder is linked to increased parenting stress and negatively impacts parental functioning and the parent–child relationship (Christie et al., 2019). The narrowing of the behavioral repertoire that characterizes pathological sequelae of trauma is highly related to mindfulness as it involves a tendency to avert from being present (Follette et al., 2006). Accordingly, trauma correlates with a diminution in mindfulness (Bernstein et al., 2011; Frohe et al., 2020). Reconsolidating birth-related trauma can reduce posttraumatic symptoms and promote parents’ mindful capacity.

Key features of evidence-based treatments for posttraumatic symptoms include psychoeducation and recounting distressing memories (Schnyder et al., 2015). Processing trauma in a safe environment helps integrate these experiences, mitigating the risk of impending psychopathology. This is crucial for parents, as birth-related trauma might be dismissed due to societal expectations that portray childbirth as uniformly positive (Horesh et al., 2021). A trauma-informed approach attests that unresolved birth-related distress can disrupt positive bonding processes and dwindle the parental space for mindful presence with the child. Encouraging open discussion and validating distress can enhance mindful engagement, promoting both parental well-being and healthy child development. Social support plays a vital role in buffering against birth-related psychopathology (La Rosa et al., 2024b) and can be fostered, for example, by enabling parents to share their experiences with compassionate others. This underscores the need to amalgamate trauma-informed elements into mindful parenting models, recognizing that healing often requires more than cognitive understanding—it demands an emotionally engaging process.

### 2.5 Making it alive

Experiential and emotion-focused approaches suggest that meaningful change occurs when individuals engage authentically with their emotions (Gendlin, 1996; Greenberg, 2008). Emotion-focused therapy posits that treatment should focus on explorations of emotional themes essential to the client to enable them to communicate more effectively, regulate arousal, and respond more adaptively (Greenberg, 2017).

When developing our approach, we reflected on personal experiences with mindfulness groups in which we participated. We noticed that sessions resonating with sincere emotional expression and deep engagement had more meaningful and even transformative effects compared to those with a non-personal, emotionally distant, or exercise-oriented approach. While mindfulness is a prominent vehicle for emotion processing, it is insufficient on its own; more profound engagement with one’s emotions is necessary for meaningful change (Geller and Greenberg, 2012; Colosimo and Pos, 2015). Recent studies have highlighted the clinical merits of integrating an emotion-focused component into mindfulness interventions (Gayner, 2019; Hatch et al., 2023). This approach holds unique value for parenting.

In mindful parenting, this perspective emphasizes practicing mindfulness amid real-life dilemmas and emotional challenges. It encourages parents to engage deeply with the charged emotions often experienced while caring for a baby. By exploring emotions, particularly difficult or marginalized ones, parents can expand mindfulness assets such as openness, acceptance, and awareness (Havighurst et al., 2020), and cultivate a more emotionally receptive parenting style, benefiting child development (Castro et al., 2015). An experiential approach can also help parents navigate destructive or painful self-positions they frequently dwell in.

### 2.6 Parental mode-work

Mode is defined as a position of the self, encompassing cognitions, beliefs, affective states, coping strategies, and bodily manifestations (Young et al., 2003). People develop predominant modes shaped by their developmental experiences. In schema therapy, a key component is mode-work, which involves identifying and giving voice to modes, facilitating dialog between them, experimenting with new modes, and modifying existing ones (Rafaeli et al., 2015).

Mode-work can enhance flexibility and awareness in parenting by helping parents recognize and modify behavioral patterns. For example, a parent might often experience self-critical thoughts such as “I’m a bad parent.” Through mode-work, this self-judgmental position can be identified and explored, enabling the parent to understand its origins and its impact on the parent–child relationship. Addressing such positions is crucial, as parents who frequently assume self-critical or withdrawn modes might unintentionally harm the parent–child relationship and the child’s sense of attachment security (Ainsworth et al., 1978). Building on a previous model (Bögels and Restifo, 2014), we propose that mode-work can increase parents’ awareness of their predominant modes, especially those that are deeply ingrained and hard to change. Raising mindful awareness of active modes in parenting dilemmas allows parents to validate the function of each mode, gage its pros and cons, and decrease the likelihood of automatically reverting to maladaptive patterns of interaction. A dialogic approach enables parents to validate and assess the effectiveness of each mode in context, exploring alternatives where needed (Burstein and Fogel-Yaakobi, 2024). This practice supports the fortification of a mindful parental mode, promoting parents’ ability to accurately understand their baby and coordinate responses to generate meaningful learning experiences through minding.

### 2.7 Learning by minding

Lev Vygotsky proposed that learning occurs within the “zone of proximal development”—the gap between a child’s current abilities and what they can achieve with guidance from a more capable agent (Vygotsky, 1978). This highlights the parent’s role, not merely as an observer but as an active facilitator of their child’s cognitive and psychological growth. Our approach emphasizes that parental mindfulness is not enough; parents must also engage in sensitive scaffolding of learning experiences.

Parents can accelerate development by accurately perceiving the child’s current skills and providing carefully tailored elaborations within their mental reach (Pratt et al., 1988; Thomas, 2013). Mindfulness remains essential to this process, as it enables parents to attune to the baby’s behavioral and emotional cues, capturing their current state—“zone of actual performance”—and offering responses that add a tip of sensation, thereby gently stretching the baby’s knowledge and abilities without overwhelming them. In these contexts, attunement refers to a mental state in which the parent resonates with the infant’s inner state—affect, arousal, and intention—enabling the parent to expand on these through subtle, contingent responses (e.g., changes in facial expression, tone of the voice, or rhythm of movement). This process is not mere imitation but rather an embodied elaborative matching of the shape, intensity, or timing of the infant’s experience (Stern, 1985). Through such responses, the infant feels “seen”, thereby supporting emotional connection and expediting deeper learning experiences within the zone of proximal development.

## 3 Theoretical synthesis

The proposed integrative framework combines mindfulness with various therapeutic approaches to offer support for parents during infancy. Each component serves a critical role in addressing the diverse emotional, cognitive, and relational needs of parents and babies in this challenging period. Mindfulness, the heart of this framework, fosters present-moment awareness and non-judgmental acceptance, which are foundational for co-regulating arousal with the child and cultivating adaptive parenting behaviors. The calming cycle theory supplies a perceptive developmental conceptualization and emphasizes the importance of mindful tactile-affective connection between parent and child. DBT complements mindfulness by promoting emotion regulation through validation and interpersonal distress tolerance skills, while trauma-informed care addresses unresolved birth-related adverse experiences that can hinder mindful engagement. Emotion-focused therapy deepens openness to experience and emotional presence, and schema therapy helps parents recognize and adjust deep-rooted and automatic behavioral patterns. Vygotsky’s learning theory further binds mindfulness to the child’s developmental growth, assisting parents to attune to and extend their child’s abilities. Together, these components form a holistic approach, ensuring that mindfulness enhances parents’ well-being and supports emotional resilience, flexible parenting, and healthy child development. Although not designed as an attachment-based framework, the proposed approach supports key processes—such as emotionally present interactions, mindful responsiveness, and co-regulation—that align with factors known to promote secure attachment. These processes, along with additional elements of the framework, contribute to fostering long-term positive outcomes in children.

Our framework builds on essential elements of mindful parenting as portrayed by previous scholars (Kabat-Zinn and Kabat-Zinn, 1997; Bögels and Restifo, 2014; Potharst et al., 2017) while adding unique dimensions to support the earliest stages of parenting. First, the calming cycle theory offers a tactile-affective lens for promoting co-regulation. Second, DBT augments mindfulness with concrete validation and distress tolerance strategies. Third, a trauma-informed approach addresses the crucial aspect of birth-related adverse experiences that can disrupt mindful engagement. Fourth, emotion-focused techniques help deepen parents’ processing of their own and the infant’s emotional states. Finally, we integrate Vygotsky’s learning theory to scaffold developmental scaffolding with mindfulness. Collectively, these additions go beyond the scope of earlier mindful parenting interventions by offering a broad theoretical base and multiple clinical applications suited to the needs of parents in their child’s first 2 years. This theoretical model lays the groundwork for the protocol presented in the following section, designed to translate these principles into a structured, accessible, and practical approach for parents.

## 4 Protocol

The following protocol translates the theoretical principles outlined above into a group program designed for parents of infants aged 3–24 months. The program integrates core global and interpersonal mindfulness skills with (1) mindful emotional presence for promoting calming cycles with the baby; (2) DBT for interpersonal distress tolerance and mitigating judgmentalism via validation; (3) trauma-informed care for processing adverse peripartum experiences; (4) emotion-focused and experiential exercises for fostering change; (5) mode-work using a designated module and consistent discussions to raise parents’ awareness and flexibility in their parenting approaches; and (6) Vygotsky’s learning theory for supporting developmental scaffolding. Each session is crafted to reflect these components, ensuring that the theoretical insights are directly applied to enhance mindful parental skills in real-world settings. We devised two versions of the protocol, one for working with parents of typically developing babies and another that addresses circumstances with an increased likelihood of stressors present—that is, following preterm birth.

The program consists of eight weekly sessions (2 h each with a 10-min break, except for Session II), guided by two certified professionals (e.g., psychologists, social workers, psychiatrists, occupational therapists, etc.) or advanced trainees with either expertise or ongoing supervision in mindfulness-based intervention and group facilitation. Each group includes 7–10 families (i.e., usually 10–16 parents). Sessions take place in a comfortable community setting, with coffee and refreshments available, aiming to create a welcoming environment.

Before group initiation, each family is invited for an individual intake meeting with both group facilitators. This meeting helps to establish rapport, discuss the family’s specific needs, and consider whether the group goals align with their expectations. During the intake phase, we establish agreements on punctuality and attendance, encouraging parents to reflect on whether they can realistically commit to the group’s demands while clearly highlighting the potential benefits of the group, such as how mindfulness can support their parenting journey. It is made clear that missing more than two sessions will result in the termination of participation, as adherence is vital for the intervention’s success. Parents are encouraged to arrive 15 min early to foster informal connections and a supportive atmosphere. Throughout the group, facilitators adopt a dialectical approach, balancing validation of parents facing adherence challenges and reinforcing the importance of consistent attendance. They also convey that every parent’s participation is valued and take steps to ensure that even quieter or less visible participants engage in a meaningful learning process that supports their growth. To augment the group’s impact, each parent is provided with an accompanying training handbook that outlines the content and exercises for each session. There are two versions of the handbook: one tailored for parents of typically developing infants (Supplementary material S1) and another for parents of preterm infants (Supplementary material S2). Detailed guidelines for group facilitators, including comprehensive session blueprints, practical facilitation strategies, and general principles, are supplied in Supplementary material S3, offering stepwise instructions and further examples to support effective implementation.

Each session sequentially builds upon the previous one, weaving the theoretical components into practical mindfulness techniques to enhance parenting skills. The general contents and exercises of each session are presented in Table 1 and below is a detailed overview of each session:

**Table 1:** Outline of group sessions.

Session	Title	Topics	Exercises	Auxiliary components	Homework
I	Establishing mindful foundations and calibrating the developmental stance	Acquaintanceship Group setting; Psycho-education on the developmental model; Core assumptions	Getting together circle	DBT; Calming cycle	None
II	Safe space for trauma-informed healing	Processing early life traumas/adverse experiences	Formative experiences from the early stages of life	Trauma-informed care; EFT	None
III	Listening mindfully	Definition of mindfulness; Mindfulness skills: (1) observing and (2) describing	Body-scanning; Mindlessness; Mindfulness video; Mindful eating		Minding when usually mindless; Mindfulness in daily routine
IV	The mindful attitude	Mindfulness skills: (3) being present, (4) non-judgmentalism, and (5) Goal-driven; Mindful moments	Mindful breathing; Judgmental vs. non-judgmental stance; Selective attention video	DBT	Observing judgmental thoughts; Taking a mindful moment
V	Applying mindfulness to parenting	Mindful attention with the child; Mindful responsiveness; Mindful interaction	Sensory awareness; Baby-mother interaction video exercise		Minding the child; Minding the child’s signals; Mindful interaction
VI	Attunement, self-validation, and emotional connection	Experience-proximal elaborations; Self-validation for parents; What parents feel is important; Parent–child mutual regulation	Validation exercises	Vygotsky; DBT; Calming cycle	Minding patterns of parent–child co-regulation
VII	Navigating distress and coping strategies	Mindful coping with distress; Interpersonal distress; Regulating distress together	Validation video; Parental modes	DBT; Schema therapy	Observing and describing interpersonal distress; Distress tolerance basket
VIII	Embracing pain and reflective closure	Embracing our pain; Summation	Summary talk (word-board)	EFT	None

DBT, Dialectical Behavior Therapy; EFT, Emotion-focused therapy.

*Session I: Establishing Mindful Foundations and Calibrating the Developmental Stance.* Session I begins with a “Getting Together Circle,” where group members introduce themselves and share a memorable moment with their child. This activity encourages parents from the outset to be mindful by focusing on a specific interaction rather than using generalizations. The session then introduces the bi-directional developmental model, which combines elements from Linehan’s biosocial theory and the calming cycle theory, inviting parents to reflect on their experiences and better understand the dynamic interplay within parent–child relationships. Interactive discussions follow, addressing foundational assumptions, which manifest a non-judgmental and dialectical approach to parental growth. The session concludes with an overview of group principles, setting the stage for collaborative and constructive engagement.

*Session II: Safe Space for Trauma-Informed Healing.* Session II focuses on creating a safe environment for parents to process adverse or distressing experiences related to pregnancy, birth, or the early postpartum period. The session begins with a 15-min guided reflective writing exercise, allowing parents to articulate their experiences. Parents are then invited—without pressure—to share their stories within a supportive group setting, where each parent speaks uninterrupted while others listen. Facilitators provide validation and gentle reframing if self-judgment arises (emphasizing how a parent did their best in difficult circumstances) and invite parents to describe any emotions or physical sensations they recall. This exploration is guided by a trauma-informed stance, enabling each parent to determine their own comfort and readiness to share while recognizing that such processing can help reconsolidate unresolved memories that are difficult to bear alone. Facilitators may share brief and relevant personal experiences with their children if time permits to model confident vulnerability and foster group trust. The session closes by acknowledging the courage it takes to explore distressing memories and emphasizing that sharing with trusted and supportive individuals can provide relief, foster connection, and benefit both the parent and the child. This structured exercise not only helps parents reconsolidate birth-related trauma but also sets the ground for an emotionally engaged group experience, thus cultivating the experiential aspect of the intervention early on.

*Session III: Listening Mindfully.* Session III focuses on developing the core mindfulness skills of observing and describing. It begins with a body scanning exercise (Kabat-Zinn, 1990) to enhance present-moment awareness, leading into a discussion on the definition and key aspects of mindfulness. The session also examines the phenomenon of mindlessness, relating it to everyday experiences in family life. The core skills are then practiced in a mindful eating exercise (Kabat-Zinn, 1990) and applied to daily routines (Nhất Hạnh, 1987) as a home assignment. This session aims to foster parents’ global mindfulness practice, helping them observe and describe their present experience as it is, without interpretation or avoidance.

*Session IV: The Mindful Attitude.* Session IV expands the foundational mindfulness skills with a brief collaborative recap of the previously learned observing and describing skills. This is followed by a mindful breathing exercise (Decker et al., 2019) to promote present-moment bodily awareness while implementing a self-regulation technique. Parents share their experience with the assigned homework, fostering peer support and group learning. The session then explores the remaining global mindfulness skills, including being present, non-judgmental acceptance, and goal-driven behavior. Non-judgmental acceptance is reinforced through a judgmental versus non-judgmental stance exercise. Next, the skill of stopping for a mindful moment (Kabat-Zinn, 1994; Kabat-Zinn and Kabat-Zinn, 1997) is discussed and accompanied by a guided home practice. The session concludes with a selective attention video (e.g., Simons and Chabris, 1999) which illustrates the concepts of auto-pilot, cultivating curiosity, and openness.

*Session V: Applying Mindfulness to Parenting.* Session V increases the focus on translating mindfulness into parenting interactions. Following a brief recap of previous skills, parents engage in a sensory awareness exercise (Selver and Brooks, 2007), such as focused auditory listening, to broaden their attentiveness to sensory knowledge. After reviewing the homework, parents are introduced to strategies for applying mindful attention with their child (Coatsworth et al., 2014), using all core mindfulness skills. This is supported by watching a still-face video, which stresses the impact of parental attention on child development. The session then explores mindful responsiveness (Kabat-Zinn and Kabat-Zinn, 1997), emphasizing the importance of balancing observation with effective responses to the child’s needs. Finally, mindful interactions (Bögels and Restifo, 2014) are discussed, encouraging parents to remain present while also caring for themselves. Home exercises are designed to assimilate mindfulness into the daily interaction with the child and enhance parents’ awareness of their child’s physiological and behavioral cues.

*Session VI: Attunement, Self-Validation, and Emotional Connection.* Session VI enhances mindful parenting by introducing a Vygotsky-based skill of parental attunement, fostering self-validation strategies, and supporting emotional co-experiences that facilitate bonding. The session begins with a discussion of parents’ experiences in practicing the assigned skills at home. Facilitators then guide parents through experience-proximal elaborations—mindful, well-timed responses that engage with the child’s present experience yet introduce new elements they can easily grasp. The focus then shifts to the role of validation in promoting well-being, encouraging self-acknowledgement and validation of emotions and behaviors as a means of mitigating self-judgment. Parents also explore how to recognize and use their emotions through a calming cycle-informed approach, fostering mutual soothing and emotional connection with their child. Lastly, parents are guided to observe and describe patterns of co-regulation with their child.

*Session VII: Navigating Distress and Coping Strategies.* Session VII equips parents with skills for mindful coping with distress and increases awareness of their predominant parental modes. It begins with a recap and sharing of homework experiences, allowing parents to reflect on practices of co-regulation and validation. The session then introduces the concept of distress tolerance (Linehan, 1993), guiding parents to identify distressing moments and explore effective personal and interpersonal strategies for moderating them. This is followed by an exploration of parental coping attitudes, promoting self-awareness and encouraging a more dialogic and non-judgmental approach to resolving parenting challenges. The session concludes with an exercise designed to help develop personalized strategies for managing distress while maintaining a mindful and effective parental position.

*Session VIII: Embracing Pain and Reflective Closure.* Session VIII begins with a brief mindfulness exercise to help parents center themselves, followed by a recap and discussion of homework, particularly involving interpersonal distress tolerance skills. The session then introduces the concept of embracing our pain (Nhất Hạnh, 2001), offering an alternative approach in which unavoidable distress is accepted rather than resisted. This discussion highlights the flexibility of navigating between actively working to change unbearable experiences and accepting difficult moments as they are, providing two complementary strategies for managing distress. The session concludes with a reflective summation of the group’s journey, where parents evaluate which skills they have successfully integrated into their parenting and identify areas for future growth. This reflective process fosters a sense of accomplishment while providing feedback that empowers parents for their commitment to improving themselves and their parenting.

### 4.1 Guidelines for protocol adaptations

#### 4.1.1 Adaptations for parents of preterm infants

Preterm birth introduces a broad spectrum of medical and developmental challenges for families, which the customized version of our protocol (Supplementary material S2) aims to address. In this context, the 3–24 months window refers to the corrected age, recognizing that many families benefit most once they have settled into home life, post-NICU discharge, and established their initial caregiving routines (Boykova, 2016). While various NICU-based programs offer transitional care (Silberstein et al., 2009b; Morag et al., 2019), promote affective touch (Silberstein et al., 2009a; Beebe et al., 2018), and offer psychosocial support (Treyvaud et al., 2019), our intervention is intended to complement these services at a point when parents have more capacity to attend sessions regularly and practice mindfulness-based skills at home. It builds on the principles of these programs to support parent-infant interactions throughout the first 2 years, ensuring the benefits of tactile-affective interventions persist beyond the neonatal stage.

It is important to note that preterm infants may still require significant in-home medical care or ongoing hospital visits during this period (Blackburn, 1995; Litt et al., 2024). For parents whose infants require more intensive care or who themselves are navigating through acute mental health challenges, specialized one-on-one support may be necessary prior to or alongside group participation. Moreover, the intake meeting is designed to ensure that parents can commit to full participation and truly benefit from the group’s practices. Through collaborative dialog, families and facilitators openly discuss whether current life circumstances support effective participation or whether alternative timing or services might be more appropriate.

#### 4.1.2 Considering personal and cultural diversity

The program’s structure and principles, including its dialectical approach and emphasis on mindfulness as a practical and flexible tool, allow facilitators to adapt discussions and practices to align with diverse populations, cultural norms, family dynamics, and community-specific needs. For example, if parents advocate self-criticism as a means of self-improvement, facilitators acknowledge such perspectives with curiosity and respect, inviting them to explore whether validation or self-validation might also be effective while emphasizing their autonomy to determine what works best for them. Similarly, some parents may approach mindfulness from a more spiritual perspective, seeking to integrate it holistically, while others hold a pragmatic worldview, aiming to gain practical tools to enhance their parenting or support their child’s development. The program accommodates both approaches, encouraging parents to explore what best suits their needs while respecting individual differences rooted in culture or personal philosophy. This approach exemplifies how the program fosters practical adaptability, permitting participants to integrate relevant skills in ways that match their values and fit their goals.

The program emphasizes inclusivity by ensuring that parents from diverse family structures can benefit effectively. While two-parent participation is encouraged, as research suggests it can lead to better outcomes (Bakermans-Kranenburg et al., 2003), the program also invites single parents to include a close relative or friend, recognizing the value of extended support systems. This approach addresses gaps in existing programs (Jeong et al., 2021), which often overlook the importance of engaging fathers or accommodating different family dynamics.

## 5 Discussion

In the current paper, we introduced a mindfulness-based infant parenting program based on a novel integrative theoretical framework addressing the needs of parents raising their young babies. By assimilating principles from calming cycle theory, DBT, trauma-informed care, emotion-focused therapy, schema therapy, and Vygotsky’s learning theory into established mindful parenting practices, this framework seeks to enhance parental functioning and foster positive bonding behaviors, laying the foundation for secure attachment during the crucial first 2 years of the child. Developing such parental skills during this transformative period in the evolution of the parent–child relationship is crucial. Previous studies demonstrated how early developmental spurts shape attention networks (Burstein et al., 2021) and emotion regulation trajectories (Geva and Feldman, 2008; Burstein and Geva, 2021), accentuating the essential role of early intervention and even suggesting that implementing the program closer to the 3-month mark rather than 24 months may be more effective in supporting optimal developmental outcomes. By fostering an emotional and behavioral parenting repertoire that reinforces mindful parental responsiveness, which is a critical factor in shaping positive intervention outcomes (Jeong et al., 2021), the program further scaffolds early development. Its short-term, group-based format, delivered in community settings, expands the scope of existing programs by providing an accessible and cost-effective approach.

Building on the principles outlined by O’Cathain et al. (2019) for developing interventions, this conceptual analysis focuses on the foundational stages, including the articulation of a comprehensive theoretical framework and the design of an integrative protocol. By constructing a framework rooted in robust theoretical bases, we strived to address the unique challenges faced by parents, including those with preterm births, while integrating clinical insights, existing evidence, and consultation with the CEO of an organization of parents of preterm children. These efforts ensure cultural and contextual relevance, which will be further refined during feasibility testing.

The current study supplies a theory and protocol, establishing the groundwork for a phased approach to evaluating the feasibility and effectiveness of the intervention. Initially, small-scale feasibility studies will estimate acceptability, adherence, and preliminary outcomes such as parental mindfulness, psychological distress, and parent–child interaction quality. These studies will also examine baseline mindfulness levels and cultural factors that may influence outcomes, using sample sizes informed by published feasibility benchmarks for mindfulness-based intervention (approximately 15–30 participants; Burgdorf et al., 2022). Insights from these preliminary studies will guide the design of subsequent larger randomized controlled trials, powered to detect moderate improvements in parental functioning, well-being, and child outcomes.

Importantly, all materials for this program, including handbooks, instructions for group facilitators, and supplementary resources, are openly available here. This ensures that practitioners and researchers can access, utilize, and clinically examine the utility of our framework, enabling them to evaluate and refine its effectiveness. By offering full access to the protocol, this study not only provides a framework for future empirical research but also underscores the importance of collaboration and accessibility in advancing intervention science.

## 6 Conclusion

The novel integrative mindfulness-based infant parenting framework presents a promising development for the fields of mindful parenting theory and mindfulness group interventions tailored explicitly for parenting during infancy, with particular relevancy to families with infants born preterm. We suggest that raising awareness in pediatric healthcare services to the possibility of incorporating the proposed protocol during this crucial period of life can present a viable means to reinforce resilience. By scaffolding early parental behaviors that support emotional connection and secure attachment patterns through mindfulness, the prospect of long-term positive child-related, parental, and interpersonal outcomes can be realized. Thus, the current framework has the potential to improve the well-being of families significantly. Our proposed theory-based protocol—provided openly and detailed herewith—calls for empirical studies to evaluate feasibility and effectiveness, with the hope that it may contribute to the rigorous and wise implementation of mindfulness-based practices in pediatric services. The open availability of the program’s materials enables transparent collaborations to promote the thriving of children and their parents.
