# Supplementary material for: Integrative mindfulness-based infant parenting program: theoretical foundations and a novel intervention protocol
Source: Front Psychol. 2025 Feb 7;16:1524008. doi: 10.3389/fpsyg.2025.1524008 (PMC11842442; doi:10.3389/fpsyg.2025.1524008)
Supplement: Supplementary file 2 [file Data_Sheet_2.pdf]

Bar-Ilan University

Mindful Parenting Group for Parents of Preterm Babies

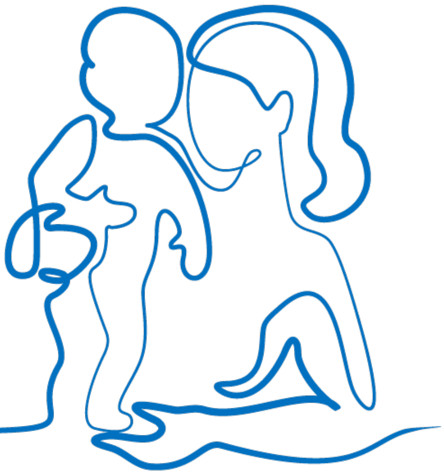

Geva Lab

Development, Social Attention and Neuropsychology

https://gevalab.wixsite.com/gevalab

Introduction

Mindfulness is an important ability that helps parents pay attention to what is happening with their children in real-time and respond effectively during interactions with the child. Parental mindfulness skills are especially important when raising infants with a tendency to restlessness and agitation, as parents of premature babies often experience. This group was developed to provide parents with mindfulness skills to help them in challenging and calm times with their baby.

The handbook accompanying the group is based on insights from many years of research in our lab and other labs around the world. It aims to provide a framework for understanding infants' emotional, social, and cognitive development while learning about risk and resilience factors. At its core stands the assumption that parents have an essential influence on shaping the development of their children.

Mindfulness skills will be the core skills we will learn and practice together in this group. Over the past decades, it has been found that mindfulness helps people dealing with various mental health issues and can significantly improve interpersonal relationships. We will learn general mindfulness skills and then focus on promoting mindful parenting skills. Mila and Jon Kabat-Zinn proposed the pioneering model of mindful parenting. The Development, Social Attention, and Neuropsychology Lab at Bar Ilan University has developed the current model by assimilating practices from the Kabat-Zinns' model, as well as other related frameworks, including calming cycle theory, DBT, schema therapy, emotion-focused therapy, and Vygotsky's learning theory (full acknowledgments are provided on the last page).

Group Goals

Main Goal

Learn and improve skills that help identify, describe, and understand the child's needs and respond effectively to support the child's development, the relationship with the child, and the sense of parental competence.

Specific Goals

1. Create a framework for understanding the child's developmental needs.
2. Promote calming cycles and strengthen our bonding to our child.
3. Promote a mindful and supportive family atmosphere for all family members.
4. Reduce ineffective parental behaviors that amplify distress levels.

Means to Achieve the Goals

1. Learn the Scaffolding-Feedback Model for child development.
2. Learn mindfulness skills related to myself, the child, and interactions.
3. Learn skills for sensitive and elaborating responsiveness to the child.
4. Expand mindful ways of dealing with parental distress.
5. Process formative experiences with the child.

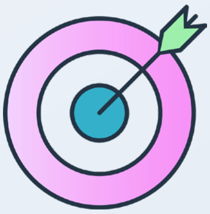

The Scaffolding-Feedback Developmental Model

The Scaffolding-Feedback Developmental Model suggests that for babies in general, especially for sensitive babies, the relationships with their primary caregivers are a vital support channel. Different babies have distinct physiological and emotional predispositions, influencing how the environment adjusts and responds to their needs. At the same time, the baby's psychological, cognitive, and emotional development is also shaped by the relationship and parental care. The effect is mutual – in both directions. Prematurity can lead to difficulties in many physiological and emotional functions, such as sleeping, eating, and socializing. Parenting a baby with sleeping difficulties can affect the sleeping hours of the parents and, in turn, may alter the vigilance and emotional state of the parents. Parental support that will help the baby develop more efficient sleep may in turn, have a positive effect on the quality of parental sleep and parents' mood, in a way that scaffolds the parent-child relationship. Therefore, according to the model, it is important to support the ability of parent and child to experience calming cycles of mutual regulation.

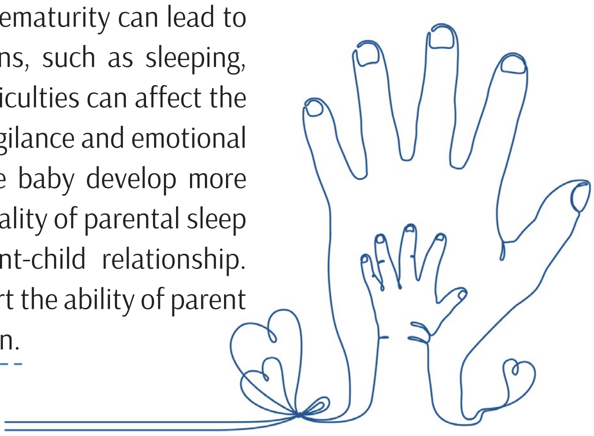

Development is carved through many small moments. When the baby is restless, it sometimes makes it harder for the environment to respond in a sensitive way. At the same time, the baby is affected by how the environment reacts. If my baby is upset and I can't calm her down, I might feel helpless, and my baby and I might grow apart, leaving her even more distressed and me with sadness. We want to support moments in which the parent manages to identify the baby's needs in real-time and respond in a way that leads to calming or joint learning.

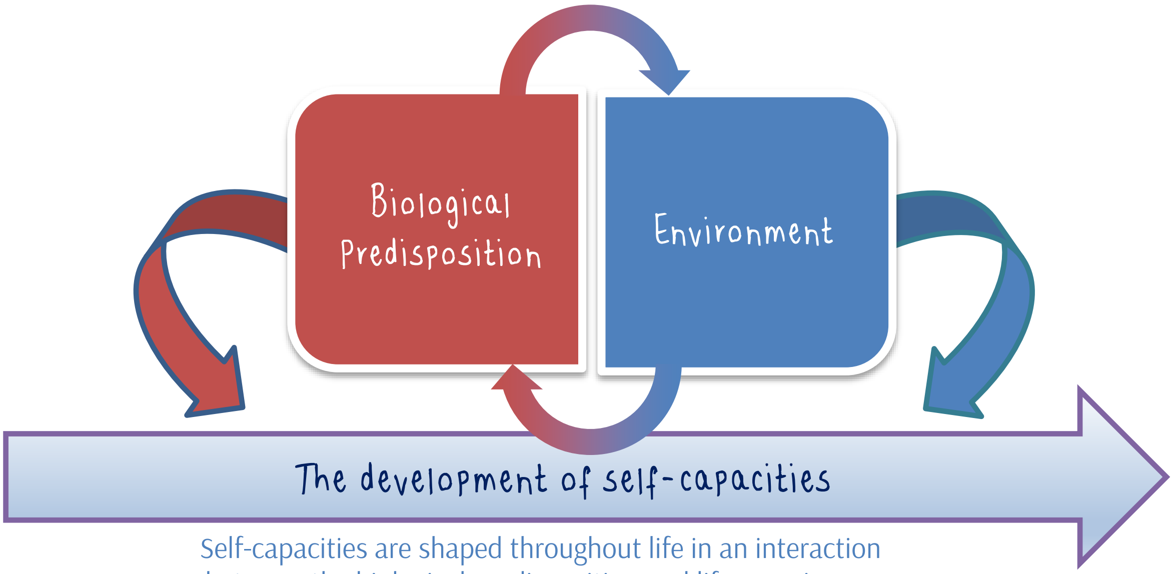

Self-capacities are shaped throughout life in an interaction between the biological predisposition and life experiences

Assumptions

1. As parents, **we do our best**, even if sometimes there are difficulties and things don't work out. In general, we believe that people do the best they can in every moment, even if they don't always manage to respond in an effective way that resolves the situation.
2. We all want to develop as human beings. As parents, **we want to improve** ourselves, the quality of our lives, and our relationship with our children. We believe that we have a natural tendency to improve and be the best parents we can be, yet sometimes, this is not enough to solve our problems. We must keep trying, get help, look for additional solutions, and practice new skills.
3. As parents, **we don't believe that it is our fault that there are problems**. At the same time, **it is our responsibility to find solutions to our problems**. We are not looking to create problems for ourselves, yet problems exist. We must take responsibility, handle the problems, and get help when necessary.
4. **Parent-child relationships are bi-directional**. Our parental behaviors affect our children, but are also affected by the child's behaviors.
5. As parents, **we are not perfect.** We accept that even if we don't want to, we sometimes act in ineffective and even adverse ways. There are reasons for such behaviors, and we need to observe them with compassion and without judgment to understand ourselves better and find new and realistic ways to respond.
6. **The lives of parents are sometimes very hard**. Our lives can be difficult (even too difficult) at times.
7. As parents, **we occasionally need support and guidance**. All humans sometimes need support, reinforcement, good advice, or simply a caring and listening ear.

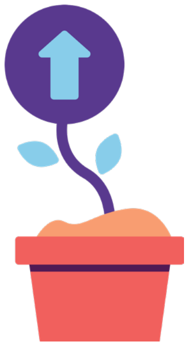

Principles for Productive Work in the Group

For the group to be productive, we will try our best to be committed to complying with the following principles:

1. Commitment to learning new skills and developing as people and parents.
2. Commitment to active learning during sessions.
3. Commitment to practicing the new skills between sessions. We will try to find an effective way for every one of you to promote an effective practice routine.
4. Maintain confidentiality and respect for privacy so that what is shared in the group remains in the group in a way that allows us all to have an experience of security and openness to share.
5. Avoid being judgmental and practice an accepting and respectful attitude toward all participants.
6. Attend all meetings and arrive on time. In case of a problem, please inform the group facilitators in advance – we, as facilitators, are obliged to try and help with every issue that arises to the best of our ability.

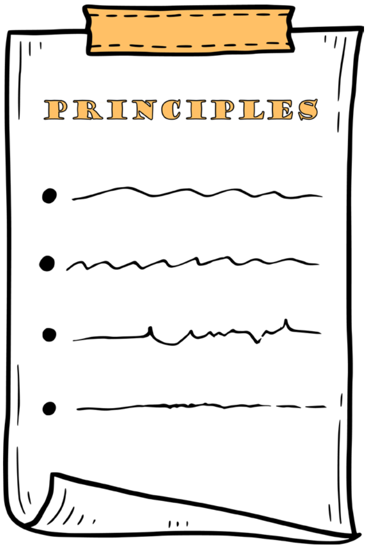

She Came Sensitive   Cheli Reuven

It's the ticking of the clock
It's the label on the back of the shirt
The smell of onion in the hectic kitchen next door
The sizzling espresso machine in the café
It's the baby (not hers) crying in the middle of the mall
The AC that suffocates the thoughts
The perfume that attacks the atmosphere
It's the grains of sand cramming between the toes
It's streets with too many cars
Places crowded with passers-by
It's the poverty of being human
The pain of existing
Everything passes through her
That's how she was born
Skinless
Nervous system exposed
It's her heart that feels
The other half of the world
The tiny crushed pea
Under the heavy matrasses
Yes, she takes everything into her heart – there she dwells,
It's the subtleties she engraves within her
The future she senses prematurely
It's the music playing in her ears
Before it was composed
It's the fuming breaths
That people exchange with each other
It's the water that gives her life
It's the wind that preserves her beauty
It's the silence she must drink
The distance
The space
That's how the world passes through her
That's how she feels everything
That's how she is – a guardian of the world

Formative Experiences from the Early Stages of Life

Parents of premature babies often experience stressful events before or at birth or while their baby is hospitalized and treated in the NICU. Stressful experiences may include a health hazard or life-threatening situation for the child or parent, exposure to stressful sights and sounds related to the child or other babies in the NICU, exposure to the child when she/he appears to be in distress or pain, and situations in which the circumstances made it difficult or impossible for the parent to treat and be with the child as she/he would want. Such experiences might cause fears that accompany us later in our relationship with the child or in life.

When we go through stressful events, we should try to observe them and share our experiences with significant people because it is difficult to bear such experiences alone. Now, we will try to recall and describe formative moments from the early stages of the child's life. We can write them to ourselves, share them with the group, or later with a close person we trust. Each of us knows the right pace of processing for them.

Describe a moment that was particularly stressful for you before or at childbirth or during the hospitalization of your child in the NICU. Try to remember what happened and <u>describe the event</u>. Write the <u>emotions</u>, <u>physical sensations</u> (tension in the body, smells, shortness of breath, etc.), and <u>thoughts</u> you experienced.

Describe a positive moment – a highlight – you experienced around childbirth or during the child's stay in the NICU. What was significant for you at that moment?

It takes courage to look at stressful or frightening experiences we've gone through. Thinking about them can bring everything to the surface and cause tension. At the same time, locking the memories inside may lead to a feeling of loneliness or to a situation where the memories seem to attack us at unexpected times and make us restless. Therefore, we will try to seek support and a caring, listening ear and use close people we trust who will be able to listen and be interested while showing us support and love. We all need this, including our child who went through the experience with us.

What is Mindfulness?

Mindfulness is intentional and non-judgmental attention

to what we feel, think, and experience in the current moment.

When mindful, we openly **accept the present moment** as it is.

Mindfulness can help us find better ways to deal with

what is happening to us and our children right now.

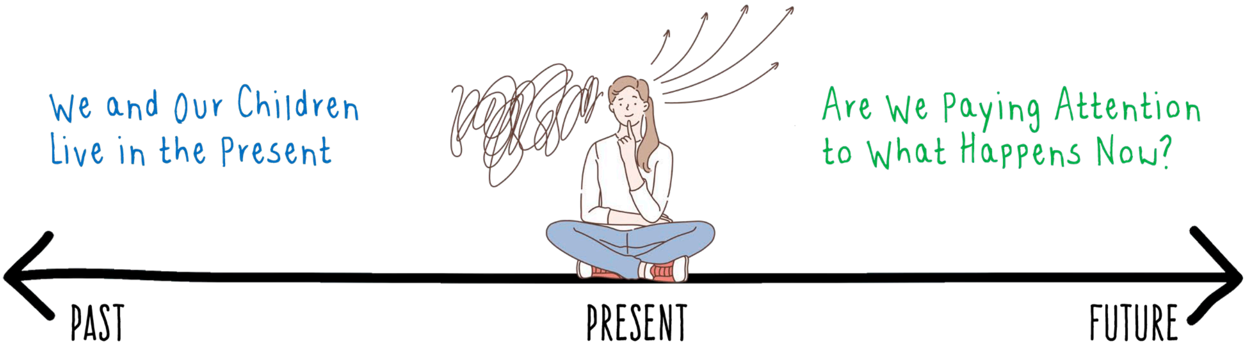

Mindfulness is the state of being aware of ourselves and our environment through **intentional**, **open**, **curious**, and **non-judgmental attention** to the **present moment**. We cannot always choose what happens to us or what we feel, think, or experience in our bodies. Mindfulness helps us to find better ways to deal with what we are experiencing now. Because trying to fight something that can't be changed can cause a lot of suffering and make it harder to find applicable solutions. Mindfulness helps us contain the present moment, recognizing that a new experience will arise in the next moment and the previous one will pass.

Mindful parenting involves the implementation of mindfulness skills in interaction with the child. Mindful parenting promotes a better understanding of what is happening now in the interaction and what the current needs of both the child and the parent are. A mindful parent self-regulates to respond wisely and effectively while recognizing the present needs. When mindful, the parent avoids automatic or impulsive responses and acts out of acceptance and compassion toward oneself and the child to strengthen the child and the relationship. Mindful parenting fosters confidence, learning, and bonding cycles with the child.

For this, we must practice the following:

- ☐ Identification of our thoughts, feelings, physical sensations, and behaviors in relation to our children.
- ☐ Minding the behaviors, physical expressions, body language, and feelings that our children express.
- ☐ Being able to respond effectively in the given moment.
- ☐ Reducing judgment toward ourselves, our family members, and our children.

Mindlessness

Being mindful is not an automatic state and usually takes practice and effort. We often find ourselves distracted, moving through our daily routines without conscious awareness. As a result, we might overlook important cues involving ourselves and those we care about. Here are several common situations that many parents experience without awareness.

Mark situations you are familiar with:

- ☐ While breastfeeding or feeding the child, you don't notice if the child is still hungry.
- ☐ You put your phone somewhere and can't remember where you left it.
- ☐ After a conversation, you can't recall what was discussed.
- ☐ Responding to our child without thinking if the response is appropriate or could benefit.
- ☐ While driving, you miss your turn because you are absorbed by other thoughts.
- ☐ You are busy with what the child is "supposed" to do and not with what is happening with your child right now.
- ☐ While reading a book, you suddenly notice that you are reading the same paragraph again and again and have no idea what you've read.
- ☐ Scrolling on your phone without any purpose.
- ☐ While playing with the child, your head wanders, and it isn't easy to stay focused.

Other situations of mindlessness you have experienced with your child:

____

____

____

____

____

____

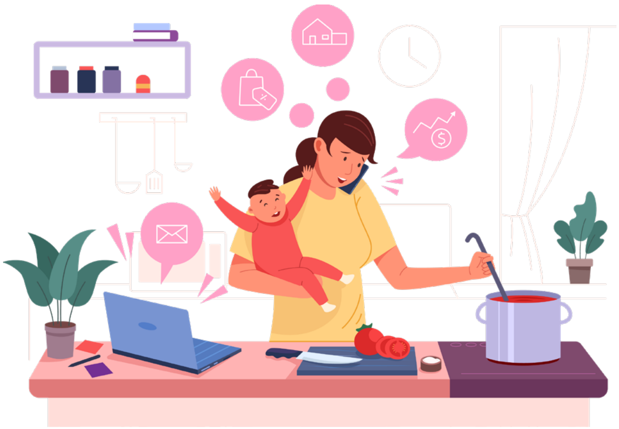

Core Mindfulness Skills

- ✓ **Paying attention** (observing, focusing our attention)
- ✓ **Describing** (conceptualizing our impressions, putting them in words)
- ✓ **Being present** (proactively staying in the situation)
- ✓ **Non-judgmentalism** (accepting the current moment as it is)
- ✓ **Goal-driven** (trying to act effectively)

So, how do we do it?

Paying attention (observing): Focusing on what is happening now: emotions, thoughts, sensory information, events, and physical sensations. Look curiously as if with a magnifying glass. Our goal is to improve our ability to focus on one thing at a time. When you bathe your child – look at her; don't read messages on the phone at the same time. Observe things intentionally without analyzing them, without judging them, and without necessarily trying to change them, whether pleasant or upsetting.

Describing: Using plain words (either in inner speech or out loud) to describe external and internal events without interpreting them. It is an ability that helps us better understand what is happening now (in our inner or outer world) and communicate effectively with the environment.

Being present: Openness to the current experience. Parents often face unpleasant feelings. There is a natural tendency to try to avoid these feelings. Sometimes, avoiding them for a short time is necessary so we do not react impulsively. But if our avoidance becomes consistent or too frequent, it does not allow us to experience life fully and prevents us from gaining meaningful emotional experiences with our children. We should pay attention to what happens even when it is sad (a shared experience of sadness with the child can be difficult, but it also teaches the possibility of processing painful emotions and can promote closeness and bonding processes).

Non-judgmentalism: Judgmentalism is any labeling of something as positive or negative, good or bad, important or insignificant. A non-judgmental position tries to see and describe the situation not as it "should" be but simply as it is. Embracing a non-judgmental stance means that we do not judge the situation at all and accept things as they are. Being judgmental can cause problems for both oneself and in relationships. Self-criticism can lead to negative emotions such as sadness, despair, or rage and harm one's self-esteem. Judgmentalism in parents can make it difficult to see what the child needs right now. Instead of grading ourselves or others or being preoccupied with what the child is "supposed" to be doing right now, we can simply observe and describe what is happening. We can learn about ourselves and our children through small-scale, non-judgmental observations. Adopting a non-judgmental attitude doesn't mean ignoring problems or glorifying our lives. Rather, it involves refraining from stamping moral tags on ourselves and others.

Goal-driven: Shift our focus to our goals and the steps needed to achieve them. Being goal-driven means prioritizing what works, not necessarily what is "right". Try to be mindful and formulate your goal for yourself at this moment. Identify if thoughts of "should" or "shouldn't" arise and make it difficult to do what could actually help in a given situation. In mindful parenting, the parent observes the situation, is open to gathering information about their feelings and the child's, and then adjusts their response to serve a purpose. Effective behavior involves modifying our prior plans or rigid rules to ensure our actions align with and support our objectives.

Home Exercise: Mindfulness in Daily Routine

Choose a daily activity (folding laundry, washing dishes, eating).

1. What activity did you choose? ________________________
2. Begin with intention. Once this week, before starting, acknowledge your intention to perform this activity mindfully. **Pay attention**. **Focus on the activity**. **Be present**. **Avoid judgment**. **Observe curiously**, as if it was the first time you were doing this.
3. Engage your senses.
   - For folding laundry: Fold intentionally; try to follow a plan. Shirts first? Socks? Feel the fabric between your fingers. Is it soft, rough, thick, or thin? Observe the colors and patterns of the clothes. Notice the odors.
   - For washing dishes: Notice the temperature and movement of the water with your hands. Watch how the water flows. How bubbles form. Listen to the sound of the dishes gently clinking. Observe each dish. Notice the scent of the soap or any residual food aromas.
   - For eating: Feel the food before eating (if it's a nut, you can play with it between your fingers). Observe the colors and textures. Feel the textures. Smell the aromas of the food. Insert the food into your mouth and observe the flavors. Chew slowly. Try to notice how the food travels along the food pipe after digestion.
4. Describe your senses. What did you notice in each sense?
   - Sight: ______________________________
   ____
   - Sound: ______________________________
   ____
   - Touch: ______________________________
   ____
   - Smell: ______________________________
   ____
   - Taste: ______________________________
   ____
5. Describe the whole experience:
   ____
   ____
   ____
6. Did you notice anything new while exercising mindfulness in your daily routine?
   ____
   ____
7. Were there moments when your mind wandered away from the task? Were you able to re-focus your attention? ______________________________

Engaging our senses in this detailed manner can transform mundane activities into rich sensory experiences. Through exercising mindfulness in our daily routine, we can strengthen our "mindful muscle".

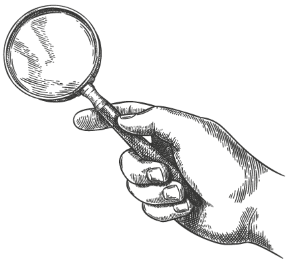

Judgmental vs. Non-Judgmental Stance Exercise

Identify an event from the past week where you were judgmental about yourself or someone was judgmental toward you. Write down what happened and focus on the judgmental aspect of the response.

____
____
____
____
____
____
____

Now, describe the situation non-judgmentally while referring to what happened, along with your feelings and the reactions and impressions you gathered regarding the event.

____
____
____
____
____
____
____

Home Exercise

Being judgmental can weaken us and the ones we love. Many of us have been frequently judged in our lives. It's not easy to change ourselves and our surrounding to become more accepting. But this goal is essential for our well-being and can be promoted through practice. In the following week:

- Observe judgmental thoughts or attitudes that you experienced. Write the judgmental voices down (what did they say?) _______________________________
____
____
____
- Notice the feelings that ensued. If you responded judgmentally to others, how did they react? If you were judgmental toward yourself, what feelings did you experience? _______________
____
____
____

Mindful Moments

Mindfulness is the ability to purposefully pay attention to our current thoughts, feelings, sensations, and behaviors. We can practice mindfulness by noticing what is happening to us at this moment. We may find ourselves thrown into situations where our reactions seem to have a life of their own, as though they are happening by themselves, and we act in a way that does not serve our goals and does not help our child to calm or explore. Such situations often occur when we raise a child with increased sensitivity or when the child is in a more turbulent period – and we are frequently exposed to intense signals of distress, restlessness, crying, or discomfort. One small moment can be the key to shaping the course of events, playing a crucial role in our ability to soothe the child and establish a sense of closeness.

Taking a mindful moment

In tense situations or those that we know may escalate quickly, or when our child really needs us, we will try to stop. Just for a little moment. We can put our hand on our leg and feel the touch; we can ask ourselves how we feel right now; we can notice the physical tension we are experiencing. Just for one moment. A brief one. Before we react. We will try to use this moment to help us choose an effective response and avoid acting on autopilot, without thinking about the consequences or what our child needs.

Home Exercise

In the coming week, practice *taking mindful moments* on some occasions. Stop for a short time and pay attention to your thoughts, sensations, feelings, and sensory input (try to choose in advance moments that you anticipate could be more challenging; for example, when leaving your child at the nursery in the morning, tucking your child to sleep at night). Try to observe with intention and without being judgmental. Be descriptive. This skill shows that using mindfulness is not necessarily a "big thing", requiring one to stop everything altogether. We want to implement mindfulness in our daily life. The more you practice this skill, the more it will be accessible to you in times of need. Pausing to take a brief mindful moment can aid in increasing awareness and fostering positive parenting habits. Fill in the following lines to describe the experience of *taking a mindful moment* (What did you notice? Was it helpful? Did you find out new things? Was it hard?)

Mindful Attention with the Child

The core skill that we want to practice and promote is mindful attention with the child. When we are mindful with the child, we exercise the basic mindfulness skills when the child and the interaction are under the spotlight of our attention (*paying attention* and focusing only on the experience with the child; *describing* the impressions we gather; while *being present* with an engaged and curious attitude; being *non-judgmental* and accepting to what this specific moment brings; trying to be *goal-oriented* and act effectively for advancing our current parental goals).

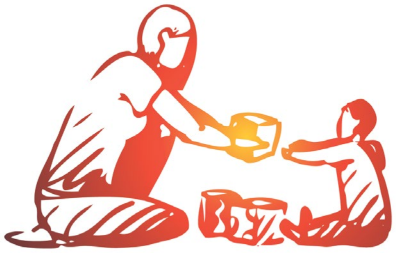

Mindfulness - meeting video exercise

Now, we will watch a short video of a baby-mother interaction. We will observe and try to stay focused on the baby and the baby only. It is natural that we'll be distracted sometimes. If this happens, we will try to gently shift our attention back to the baby.

Describe the impressions you gathered regarding the baby we observed. Focus only on describing the overt behavior without adding interpretations or drawing conclusions. Discuss the behaviors you noticed in the video separately from the feelings you experienced while observing.

Minding the child home exercise

Choose a moment in the coming week when your son/daughter is relatively calm or tranquil (playing on the mattress, bathing, or taking a stroll in the garden). For three minutes, observe the child with curiosity.

Describe the impressions you collected regarding your son/daughter. Focus only on describing the overt behavior without adding interpretations or drawing conclusions. Discuss the behavior you noticed in your son/daughter separately from the feelings you experienced while observing him/her.

Mindful Responsiveness

A mindful response is a sensitive and effective parental response that acknowledges and addresses the current needs, feelings, emotions, and expressions of the parent and the child, identifies them and describes them in a non-judgmental manner, and responds to them in a way that aims to meet physiological and emotional needs or promote joint calming or learning.

So, how is it done?

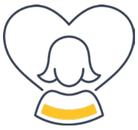

Minding Myself.

To respond mindfully, parents must also pay attention to what is happening within themselves – what they are experiencing emotionally and physically. This is important! Paying attention to ourselves also helps us take care of our children. We will remember to tell ourselves that everything we feel is understandable and normal in the specific circumstances.

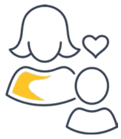

Minding the Child.

Observe and describe the situation. Pay attention to the motor and facial expressions, the body language, the verbalizations, and the words he/she says (if he/she is already communicating with words). Most of the signals that small babies convey are through the body. This is their way of explaining how they feel, what they need, and what calms them or causes them distress. We will try to observe the child with openness, curiosity, and engagement, focusing only on him/her and what he/she is expressing right now (and not on what was yesterday or maybe will be tomorrow). For a few moments, **we will try to look at the child as if it was the first time we met**.

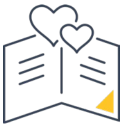

Connecting Prior Knowledge.

We can learn about our children from previous interactions. For example, a smile with rapid up-and-down hand movements in the past signaled to us that the child likes the food we gave him to taste, and weak and recurring moans around bedtime previously signaled tiredness. We can use our existing knowledge regarding the child while keeping in mind the current circumstances (where we are, the time of day, noise in the environment, current health conditions of the child, etc.), general developmental knowledge (pains around teething, children's need for cuddling, children's need to play and explore, etc.), and how they may influence the situation.

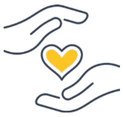

Trial and Error.

After we have been mindful of ourselves and our child and have considered our prior knowledge, we will try to respond effectively to the situation. But... this dance does not end here, as life is always in motion. It is possible, for example, that the signal that the child usually used to tell us that he/she was hungry was, this time, trying to tell us something different and new. Therefore, we must act to the best of our ability and then... **stay mindful**. Paying attention to whether our response "hit" or "miss". We will be open to relearning, updating the signal translation, and adjusting our responses accordingly. Through trial and error, we can develop new knowledge regarding our parenting and child and avoid rigid and ineffective reactions.

The main goal of mindful responses is to support the child's development and strengthen our relationship with the child. Mindful responses can address physiological needs (food, sleep, dealing with physical pain) or emotional-developmental needs (touch, security, play, and exploration of the world). In mindful responses, the parent also matters! Sometimes, the most effective thing for the child will be a parent who takes care of themself and eats – for example, by eating, a hungry parent can calm and enable themself to play with the child attentively.

Minding the Child's Signals

In the coming week, observe and describe signals you have noticed in your child around key moments. Describe the position of the body, movements, facial expression, gaze direction, and sounds the child makes (and more...) in these situations:

Sleep. Observe your child near bedtime (easier if there is already a relatively stable bedtime hour, for example, after bathing in the evening). Describe the signals you identified.

Eating. Try to identify a moment when your child tried to signal to you that he/she was hungry. Describe the signals you identified.

Bowel movement. Try to identify a moment when your child showed signs that he/she needs to poo. Describe the signals you identified.

Touch. Observe your child during a moment of physical contact between you. How did the child react to your touch? Did the child show signs that the touch was pleasant or soothing? What were they? Did you notice signs that your child wanted to change position or that a certain touch was less pleasant for him/her? What were they?

Mindful Interaction

Parent-child interaction is an important factor that shapes development. Through shared experiences, during appropriate time windows, parents can foster emotional connection with their child and promote meaningful learning: learning words (when the parent names an object that he/she and the child explore together), learning of features and characteristics (for example, smells or sights that characterize different objects), learning about the effects of motor actions on the environment (for example, discovering that a toy car continues to roll after being gently pushed or that a music box makes sounds when rotating the handle), and much more. Through mindful interactions, the parent can help develop the child's independence and learning ability. So that these joint moments will serve as a driving force for development, the mere physical presence of the parent is not enough. An active and deliberate emotional presence is also required. In other words, parents need to provide their children with significant experiences in which they offer their minds, bodies, and hearts while playing together.

Mindful Interaction Exercise

- ☐ While the child seems to be calm (for example, after eating or sleeping well; basic physiological needs in children need to be met before playing time), we will proactively decide to designate 5 to 10 minutes in which we are with him/her and only him/her (no phone, no other tasks).
- ☐ We will settle together on the carpet in the living room, on an activity mat in the garden, on our knees, or in any way that is comfortable for him/her and us.
- ☐ We will present a limited number of stimuli (toys, or if we are in nature, we can simply use leaves or pinecones in the environment). It is important not to overwhelm and that the environment will be inviting and enabling.
- ☐ Now... let things happen by themselves when we apply the mindfulness skills we learned and focus on the child.
- ☐ We will pay attention to the child. To his/her gaze direction. To movements. To the support he/she needs from us (for example, physically scaffolding a toy that eludes him).
- ☐ We will try to use mindful responses in which we support the child's investigation
- ☐ with curiosity and without trying to control it.
- ☐ We will try to have a daily mindful interaction with our child.

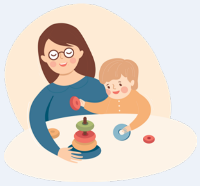

Observe a mindful interaction you had with your child this week. Describe the course of the interaction (without interpretations). Refer to what happened, what you recognized in your child's body language, facial expressions, motor behavior, vocalizations, and gaze direction, as well as the reactions and feelings that arose in you.

Experience-Proximal Elaborations

Developmental psychologist Lev Vygotsky proposed that to help our child learn, we must direct our responses so that they are within reach – in what he called "the zone of proximal development". Each child has a certain ability in the present, which determines what he/she can learn at a given time and what will be beyond reach. To demonstrate the idea, let's imagine that someone teaches us a new language with which we have no prior knowledge (current ability): she could simply speak to us fluently in this language, and we wouldn't understand anything (beyond the zone where learning can occur) or teach us word by word when displaying in front of our eyes an illustration of the new word (and then we can absorb the word because it is in the current zone of our proximal development). The argument is that a parent or any other figure who wants to succeed in teaching something should direct their responses to the zone of proximal development; otherwise, learning will not occur.

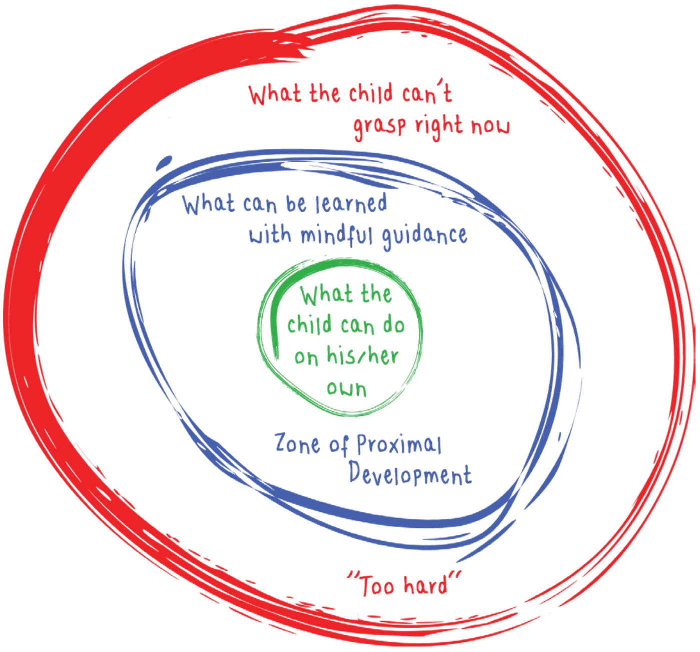

What is Experience-Proximal Elaborations?

Parental responses that are attuned and refer to the child's current behavior and add to them a layer close to the child's experience (in the zone of proximal development). They are carried out from an open position that involves a degree of playfulness. Through such responses, parents can teach their children new things. The most important skill for experience-proximal elaborations is mindfulness because it is only through active attention that we can understand what the child is currently experiencing and what can be elaborated.

Types of Experience-Proximal Elaborations (based on Daniel Stern)

- **Change in Shape.** Reactions in which the parent refers to the child's behavior and responds through another sensory channel (for example, the child drives a toy truck, and the father adds the noise of a rattling engine - the addition of an auditory channel to the experience of touch and sight).
- **Change in Intensity.** Increasing or decreasing the intensity in the same sensory channel (for example, repeating the baby's speech, but more loudly or in a different tone).
- **Change in Time or Rhythm.** A response that plays and changes the pace of the original response (for example, the parent repeats the child's movement but performs it more slowly).
- **Combination.** Sometimes elaborations include changes in several ways (for example, shape and rhythm) and several senses simultaneously (for example, sight and hearing).
- **Naming.** The act of uttering a word. This is how children acquire language. Not every naming is an elaboration. We want to teach words in a way that follows the child's current exploration and adds a change in shape to what the child is currently experiencing. For example, when the child is hit by the toy and feels pain, we can bring our hand closer, caress the stricken area, and subtly say, "Ohh... it hurts", thus teaching our child the concept of "pain" or the word "hurt".

Self-Validation for Parents

Validation is the act of communicating to others that their responses make sense and are meaningful within a specific context of life. That, however the others behaved or whatever they felt is understandable, given the circumstances.

Self-validation is the ability to affirm to ourselves that our feelings, thoughts, and behaviors are understandable and stem from valid reasons in a certain situation. Validation is necessary to aid us in learning to acknowledge our emotions, recognize our experiences, and trust ourselves. It is the ability to confirm to ourselves in a non-judgmental manner, from an accepting and compassionate position, that what we feel, think, experience, or do is important.

Validation does not necessarily mean we agree with what we do, say, or feel. Sometimes, we all act in ineffective ways that cause pain and distress to ourselves and our close ones. Surely, we don't like such behaviors and don't want to endorse them. In validation, we suggest that even harmful or ineffective behaviors have valid reasons. And if we try to understand these reasons while acknowledging what we felt, thought, and experienced in the specific case, we might find wiser ways to deal with tough situations.

How do we do it?

Be Mindful.

Identifying and describing the experience as it is. Describing without judging or adding interpretations ("I feel lonely"; "I'm anxious")

Accept.

Accepting that what we feel or experience is valid. It doesn't mean we are comfortable with the emotion or want it to linger; it simply means we accept our feelings. It involves affirming to ourselves that, given the circumstances, our experience is understandable ("It makes sense that I feel anxious right now, given the medical procedure my son is supposed to undergo tomorrow"). In tough situations, when our attitudes or actions might be counterproductive, even then, it is crucial to understand what triggered us to feel or behave this way.

Being mindful and accepting can change the way we treat ourselves and the way we react. To achieve that, we must take ourselves seriously and not dismiss feelings and emotions. And avoid being judgmental, especially when our behavior is not ideal. In self-validation, we show compassion for ourselves and acknowledge that things happen for a reason.

We believe that self-validation is not enough. We also need meaningful people in our lives who can give us experiences of being validated. We need this as parents and as human beings.

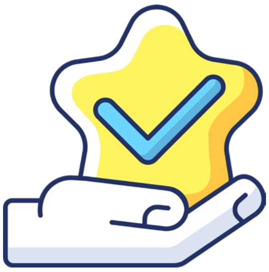

What Parents Feel is Important

Parenting is filled with positive and empowering experiences that bring meaning to our lives. However, sometimes it also comes with challenging moments and painful emotions that can be difficult to endure. It's not easy when it happens. Parents often think that the baby should not be exposed to them when they experience negative feelings and, therefore, should avoid or withdraw from the baby. But usually, we don't choose how we feel, and our children need us even when we feel bad. When we avoid or try to fight the emotion, we might make our child feel less secure with us. We want to make room for our feelings in an appropriate way that does not frighten or overwhelm the child (it is likely that if we yell at the child when we are desperate, it will not calm anyone down).

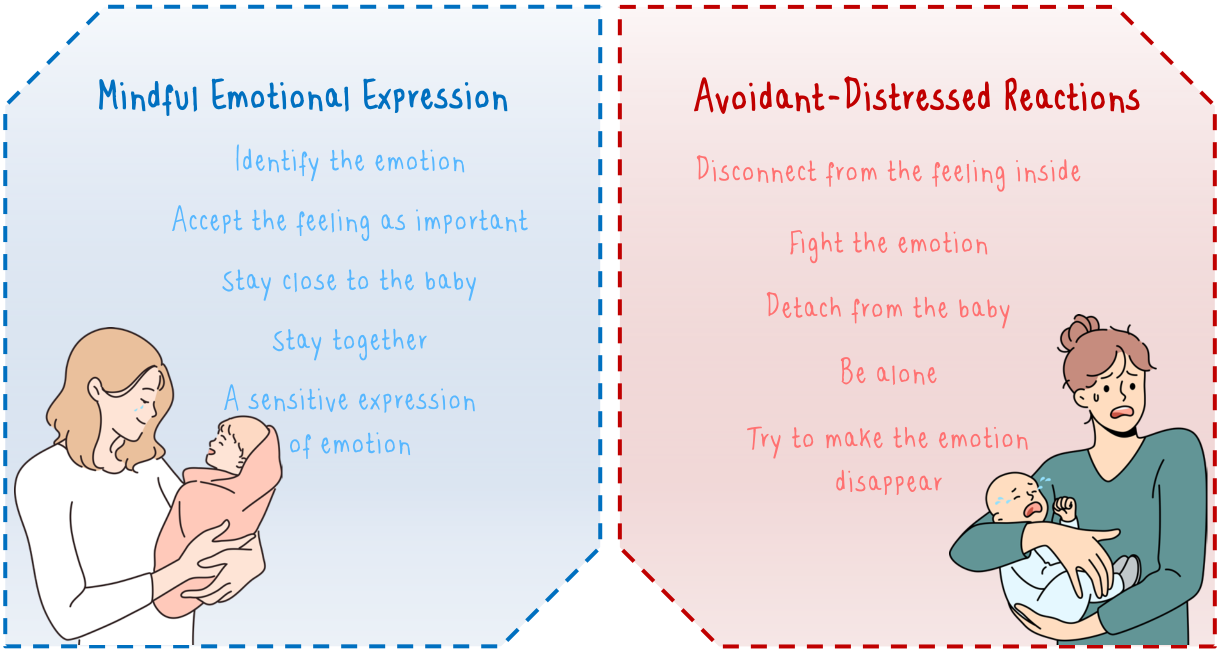

What parents feel is important! We have good reasons to feel what we feel. It is important that we learn to pay attention to our feelings and believe that they come to teach us something about ourselves and the significant relationships in our lives. We don't necessarily have to like what we feel, but we should try <u>to pay attention to the emotion</u>, <u>accept that it is real and ourselves when we feel it</u>, <u>and understand that what we experience makes sense in the specific circumstances</u> (*"It makes sense that I would be sad, worried, and exhausted right now when the child is not calming down and I have been alone with him for so long already"*).

It's important for parents to make room for what they feel. The idea is that no emotion will be a barrier to the relationship. It is sometimes challenging with negative emotions. Babies need their parents with them even when the parents are sad. We want to be able to stay close even when it's hard to feel what we feel. Sometimes, it can help to verbally express the emotion while maintaining physical proximity and contact with the child without necessarily trying to change the emotion or the situation. For example, in a moment of despair, sorrow, or loneliness, we will try to pay attention to our experience with acceptance and self-compassion, we will get closer to the baby or pick her up and hug her, and we can subtly say, *"Mommy is really sad right now my dear"*. Such responses help to promote mutual regulation of the baby and the parent. There is immense power in being able to stay together during tough emotional experiences. It can strengthen our bonding instincts to our child and teach us that we can be competent even when we feel down.

Parent-Child Mutual Regulation

The idea of mutual regulation comes from the assumption that moments of distress or tranquility occur in the relationship between a baby and a parent in real-time. This is different from previous concepts that looked at the emotional development of the baby as standing on its own. According to the mutual regulation approach, a moment of distress can start with the baby (hunger, tiredness, physical pain, etc.) or with the parent (hunger, fatigue, loneliness, sadness, despair, fear, etc.) and then almost inevitably affect the interaction and the other side. As parents, we can be mindful of moments of distress that arise and try to respond in an effective manner that helps us nurture cycles of joint calming. The ability of parents and children to calm together is very important for the baby, the parent, and the relationship. Below is an illustration of a calming cycle in which a mindful response of the parent helps the parent and the child regulate the distress levels together.

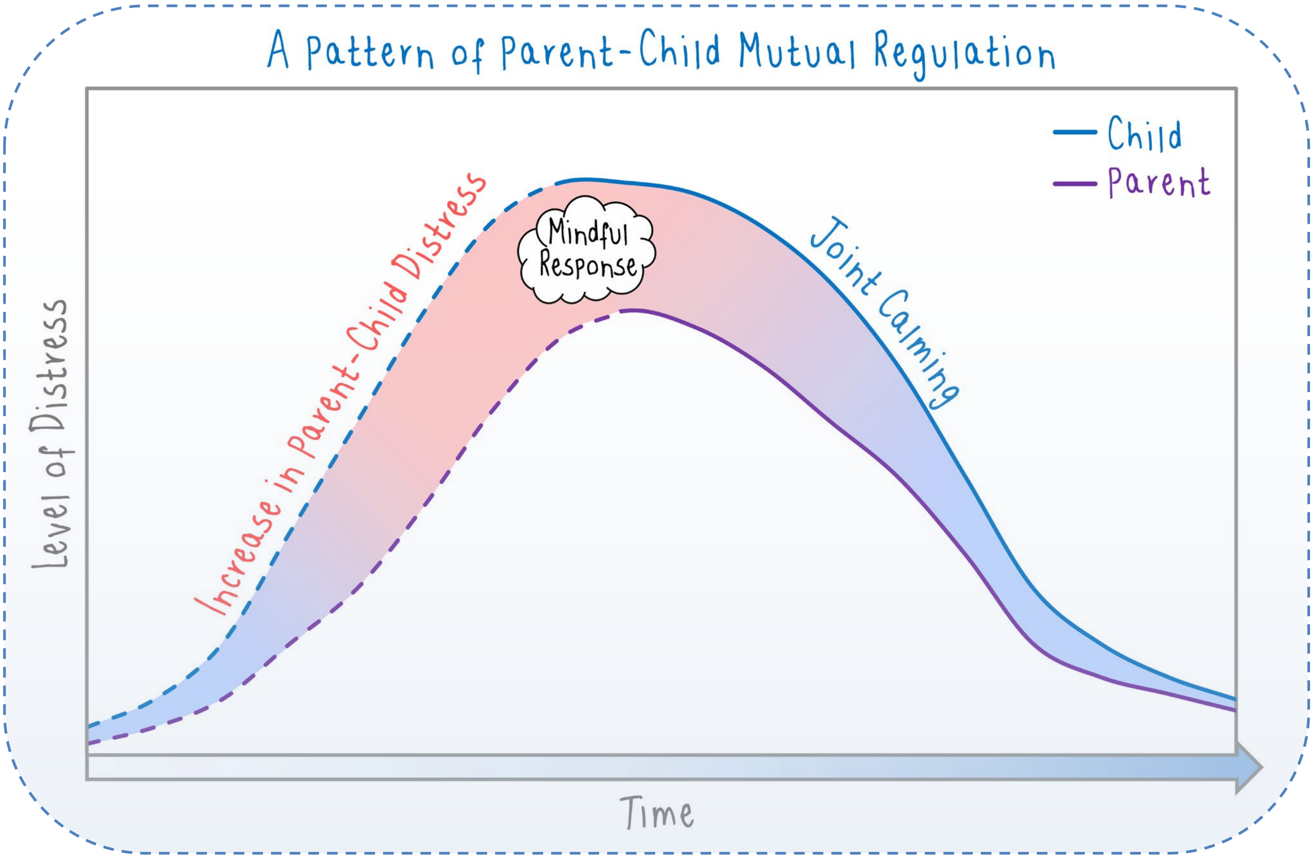

Describe an event of distress that you experienced together with your child in the past week. How did the incident begin (did the initial distress arise in the child or you)? How did the distress affect the other side (how did the child react to your distress, or how did your child's distress affect you)? How did you react? What were the outcomes of the reactions (did you calm together, or maybe the distress actually increased)?

Mindful Coping with Distress

We all have our share of stressful and even unbearable situations. As parents, we might experience moments when we are wearied and sad, and the baby continues to cry on and on. Mindful coping with distress is necessary because there are situations that we can't change or can't change the feelings we have during them. Anxiety, sadness, and pain are all a part of life and can't always be avoided. When the negative emotion is "too much", we might react in harmful ways that do not serve our goals. We want to promote effective ways to deal with distress so that we can overcome adversity together with our children while strengthening ourselves and them.

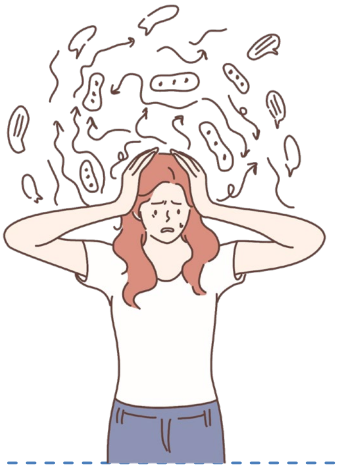

Describe a recent incident in which you experienced distress while taking care of your child. How did you deal with this incident?

The event:

How you dealt with the distress:

What were the outcomes of this coping strategy?

Interpersonal Distress

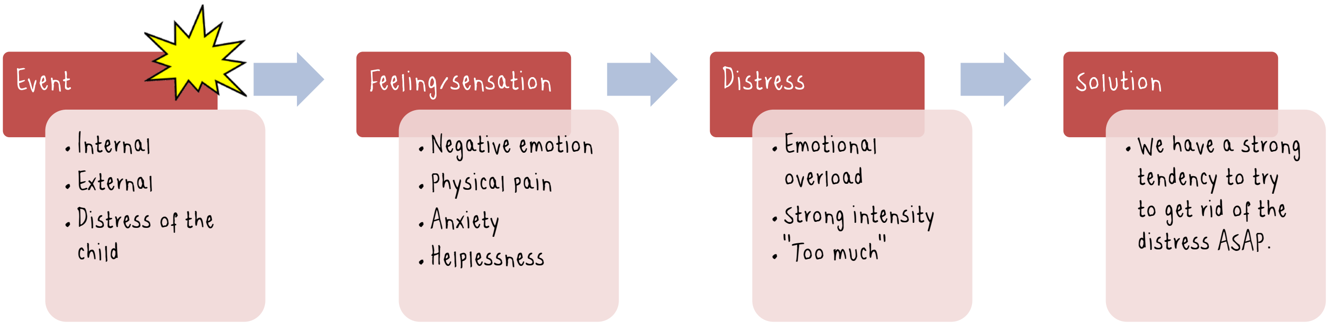

Stressful or distressing situations have common characteristics and are shaped dynamically by what happens to us and our children at a given moment.

Interpersonal distress events can start from the distress of the child or difficulties related to taking care of the child (prolonged crying that does not subside, physical pain of the child, a complicated medical condition, etc.), which leads to an intense adverse emotional or physical reaction of the parent (anxiety, shame, rage, tension in the body, despair). When the intensity of the aversive experience is too high or unbearable, we experience distress. Events of distress with our child can be awful. It is natural to try to get rid of such an experience immediately, even if the response might be counter-productive (for example, yelling at the child so loud that he becomes silent in fright). Such behaviors harm relationships and the child's ability to self-regulate. We must do our best to promote effective ways of regulating distress with our children.

Mindful coping with interpersonal distress:

1. Coping from a position of **acceptance** of ourselves, the child, and the situation without being judgmental.
2. Withstanding the distress **without harmful parental behavior**.
3. Being able to take a **mindful moment** for effectively regulating parental distress. We first aim to decrease the distress level from the "OVERLOAD" region so that we could, in the end, experience a positive moment of joint calming with our child.
4. In distressing situations, even **temporary relief** is important. We don't need to solve all our problems at once. Especially when our current state of mind is less equipped to deal with it.

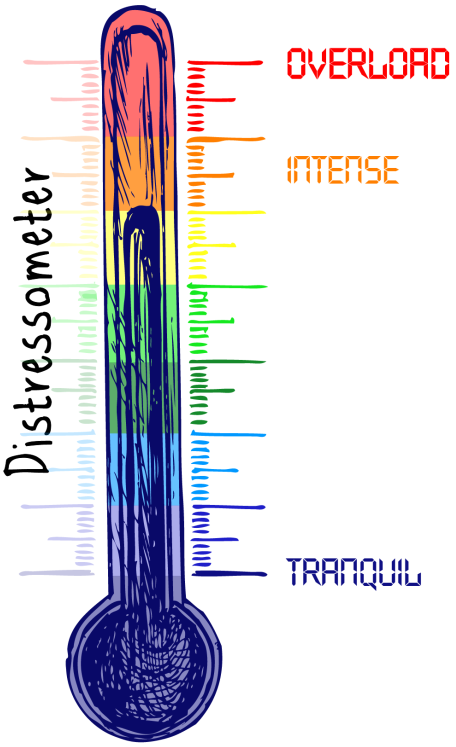

Coping Attitudes of Myself and My Parents

As parents, we face distressing situations with our children and have several core strategies we usually use to deal with such situations. Interestingly, the cycles and the coping attitudes we experienced as children with our parents can influence the core coping attitudes that we developed ourselves. Now, we will try to observe how our parents responded to our emotional needs when they were in distress with us.

Try to recall a distressing event you experienced with one of your parents as a young child. Describe the event and consider the following: (1) What were your emotional needs? (2) How did your parent react? (3) Were your emotional needs met by the parent? (4) How did you respond to your parent's reaction? Did you feel better? Worse?

____

____

____

____

____

____

____

Mark the sentences that describe the typical reactions of one of your parents during times of distress with you when you were a child:

___ Was very harsh and strict.
___ Used physical punishment or harsh discipline.
___ Was unpredictable in his/her reactions.
___ Paid attention and made an effort to understand how I felt.
___ Blamed me.
___ Made me feel like I couldn't rely on my judgment.
___ Withdrew from contact with me and left me all alone.
___ Made me feel like there's something wrong with me.
___ Showed warmth and affection toward me.
___ Shouted at me.
___ Was emotionally cold.
___ Did not set limits even when it was necessary.
___ Was open and curious to whatever I had to say.
___ Made me feel like I'm important.
___ "Self-sacrificed" himself/herself; tried to take care of me but with me feeling bad as if I'm harming his/her health or making him/her feel bad.

Other coping attitudes my parent used with me:

____

____

Are there any attitudes your parents used with you that sometimes (whether you like or not...) you use today? Every coping strategy tries to solve something – what do you think the goal of this strategy is? Is it effective?

____

____

____

____

____

Regulating Distress Together

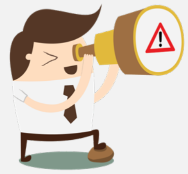

Identification

The **first** and **most essential step** to deal with distress effectively is to recognize that I'm in distress. Remember situations where I'm more vulnerable (in the morning, when I'm tired, days when I'm completely alone with the child). If we manage to stop for a mindful moment and notice that we are in distress (or that our baby is in distress), we have better chances of finding effective ways to cope.

After identifying that we are in distress, we can learn and exercise various coping methods. The following skills for mindful coping with distress are presented like a row of groceries in the supermarket. The skills on this page are primarily oriented to help us decrease the distress beneath the critical level (from *OVERLOAD* to *intense* or below). When we are in OVERLOAD, it is okay to find temporary relief by distracting ourselves, generating an alternative experience, or soothing ourselves through positive sensory experiences.

The main goal is for each and every one of us to try out and collect a personal basket of skills that are effective for us. It is also possible to collect other skills that do not appear in this specific row... Try to talk and think about practical ways of coping for you with your spouse or a close person.

Remind yourself that distressing situations are challenging to cope with, and, understandably, sometimes, the responses will not be ideal.

There are individual skills and mutual skills that can be exercised with the child. It is also important to collect mutual skills that can help us soothe together with the child. However, there are situations where it is more effective to calm ourselves first.

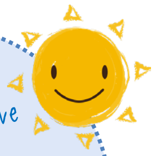

- # **Give this moment a meaning.** Try to find a meaning for enduring the pain you're going through. Give yourself a purpose for enduring this moment.
- # **Think about something else.** Count yellow objects in the room. Think of worse situations in life. Name as many classmates you can remember from your primary school. Plan your next vacation.
- # **Breathe mindfully.** Exercise mindful breathing.
- # **Use your imagination.** Try to imagine a pleasant experience you had recently. Try to visualize a person who usually soothes you or a place where you feel safe.
- # **Say kind words to yourself.** Encourage yourself. Remind yourself that you are doing the best you can and that you are GREAT.
- # **Maintain a half smile.** Let a half smile subtly come.
- # **Seek validation or support.** Call or talk to someone who can offer you validation or support.
- # **Use your senses to create an alternative experience.**

    Touch. Hug your child. Give yourself a 30-second hug or ask your partner for a 30-second hug. Play with water with the child. Do an activity of coordinated movements with the child. Bathe your child. Take a hot and comforting bath.

    Sight. Take a stroll in the park with your baby. Look at pictures together. Observe your child.

    Hearing. Listen to a song you love or sing a song to your child. Listen to sounds that calm you (water tapping, wind, rain, birds, waves in the sea) - you can also do it in the park.

    Smell. Smell the odors of different fruits or other pleasant stimuli with your child. Experience the body odors together with the child.

    Taste. Eat something delicious. Drink your favorite beverage. Buy yourself a food that you will enjoy eating.

Practicing Mindful Coping with Distress

Which of the following skills have you used in the last month during a moment of distress while caring for your child?

Give this moment a meaning; Think about something else; Mindful breathing; Use your imagination; Say kind words to yourself; Maintain a half smile; Seek validation or support; Create an alternative sensory experience.

Circumstances of the distressing event	Skills you used	Outcome

Homework

Build a personal basket of skills to deal mindfully with distressing situations. Try to recall the skills mentioned earlier that you think could help you or other skills that have helped you cope effectively with distressing situations in the past. Write down the skills on the page. You can use these skills when you encounter a distressing situation.

Try to think of specific skills that especially help you and your child deal with distress together.

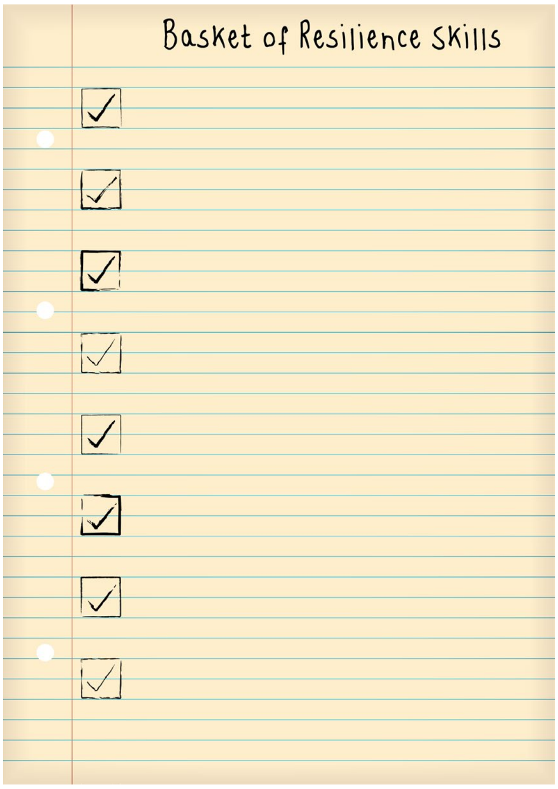

Embracing Our Pain

The idea of embracing our pain is based on mindfulness practices of recognizing and accepting things as they are and not as they should be. There are moments when pain is unavoidable. The strategies we discussed earlier can help us alleviate distress by distracting our minds or creating an alternative and stronger experience. Along with these strategies, there is another possibility of embracing the moment, however painful it may be. When embracing pain, we do not try to change things; we rather observe the situation, recognize what happens now – how we feel, what our body experiences, how our child behaves – and accept it. Even if it is painful or even too painful. This idea may seem counter-intuitive. We have a strong tendency to try and fight a situation that seems unbearable. However, fighting adversity that can't be changed might make things worse. When embracing our pain, we let the pain wash us entirely and then... pass. Zen master Thich Nhat Hanh suggested the metaphor of *making your unwanted guests feel at home* to describe this position. Looking straight at our "unwanted guests", describing them, and accepting whatever they may bring can make them smaller and relieve our pain faster.

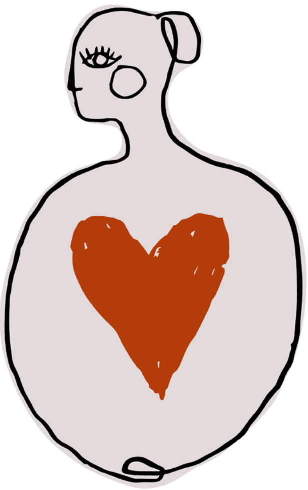

So, how is it done?

Recognizing. Staying present, paying attention, and describing the current experience. The despair, sadness, tension, anxiety, fear, anger, loneliness, physical pain, jealousy, fatigue, or restlessness. Recognizing all aspects of the experience, including our emotions, thoughts, bodily sensations, and the reactions of our child.

Embracing. Accepting ourselves and our child in the current painful situation. Rather than fighting it, we embrace it. Rather than being judgmental, we accept the current reality. When feeding doesn't go as planned, and the parent becomes nervous that the child did not eat enough, and a frightening thought arises having to do with possible underlying health issues of the child – the parent embraces the anxiety ("*I see you Fear. It's OK that you're here. You have your reasons for coming. You're welcome to stay, and I'll do my best to take care of myself and my baby while you're here*").

Choosing not to fight with our pain does not mean we stop making efforts to find new solutions to our problems. When we assume a mindful position toward our pain, we can make room for change by recognizing and accepting it. It's difficult to change something we don't acknowledge. When we embrace the current reality instead of fighting it, we can often find better ways to deal with the issues at hand.

As parents who apply the principle of embracing our pain, we create experiences with our children that teach us together that pain is a part of life and that it is possible to survive painful experiences while remaining close to each other.

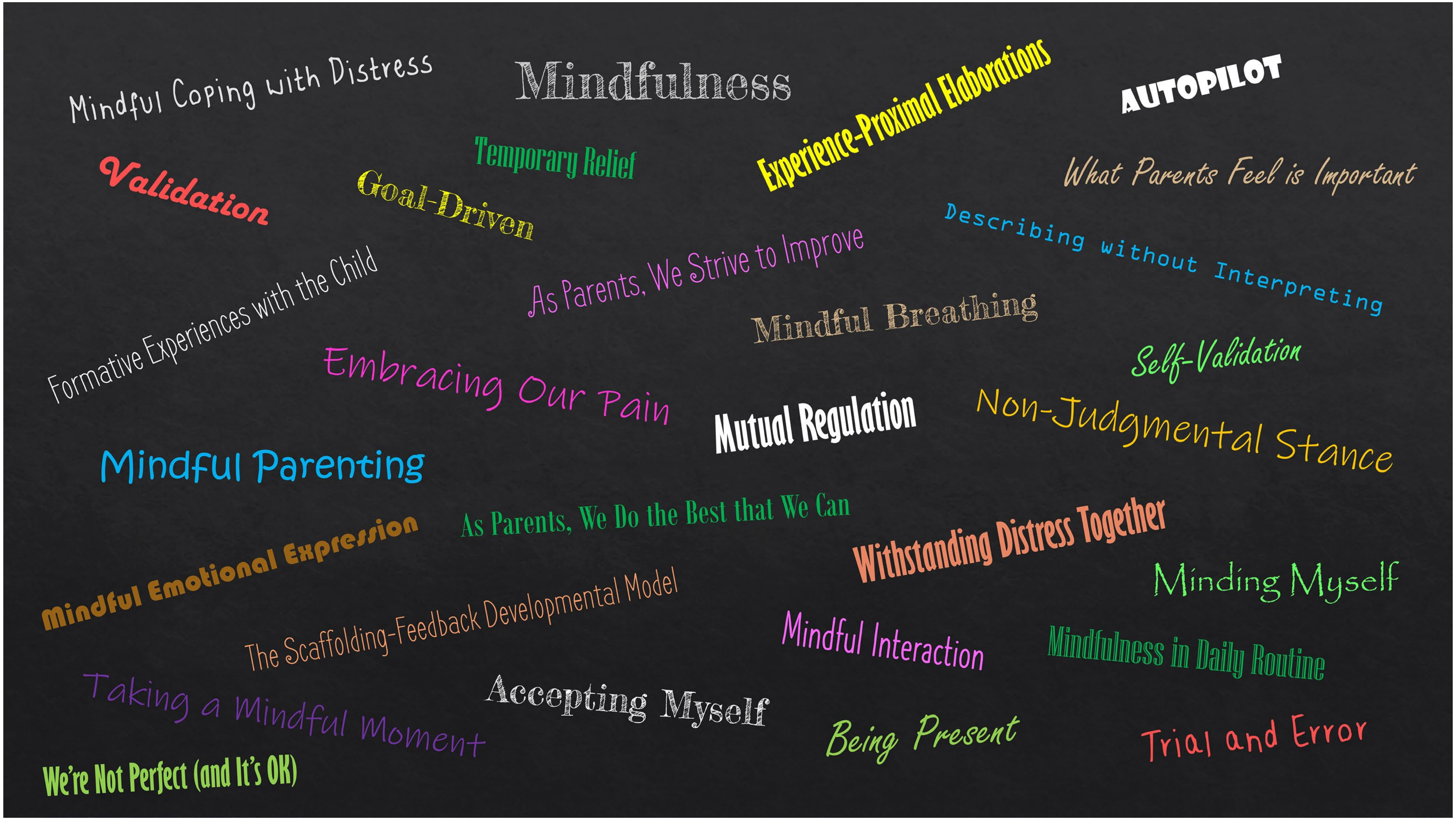

Observe the word board with the main concepts and skills from the group. What is the main skill that you implemented in your daily life? What skill do you want to improve in the following year?

Acknowledgements and Further Reading

For further details on the intervention, theoretical discussions, and personal thank you notes, see:

Burstein, O., Teshale Zevin, Z. & Geva, R. (2025). Integrative Mindfulness-Based Infant Parenting Program: Theoretical Foundations and a Novel Intervention Protocol. *Frontiers in Psychology*. doi:10.3389/fpsyg.2025.1524008

Below, we present a list of references to books, articles, and materials that have influenced, inspired, and shaped the development of this program. We extend our heartfelt gratitude to the pioneering clinicians and researchers who have advanced the field of mindful parenting, providing the foundational steps upon which this intervention was cultivated. We also acknowledge the complementary practices and developmental theories that have enriched our understanding and further enhanced the scope of this program.

Acknowledgements

Bogels, S. & Restifo, K. (2014). *Mindful Parenting: A Guide for Mental Health Practitioners*. Springer.

Duncan, L. G., Coatsworth, J. D. & Greenberg, M. T. (2009). A Model of Mindful Parenting: Implications for Parent–Child Relationships and Prevention Research. *Clinical Child and Family Psychology Review, 12*(3), 255–270.

Greenberg, L. S. (2017). *Emotion-Focused Therapy: Revised Edition*. American Psychological Association.

Kabat-Zinn, J. (1990). *Full Catastrophe Living, Revised Edition: How to Cope with Stress, Pain and Illness Using Mindfulness Meditation*. Delacorte Press.

Kabat-Zinn, J. (1994). *Wherever You Go, There You Are: Mindfulness Meditation in Everyday Life*. Hyperion.

Kabat-Zinn, M. & Kabat-Zinn, J. (1997). *Everyday Blessings: The Inner Work of Mindful Parenting*. Hyperion.

Linehan, M. M. (1993). *Cognitive-Behavioral Treatment of Borderline Personality Disorder*. Guilford Press.

Nhat Hanh, T. (1987). *The Miracle of Mindfulness*. Beacon Press.

Nhat Hanh, T. (2001). *Anger: Wisdom for Cooling the Flames*. Riverhead Books.

Rafaeli, E., Maurer, O. & Thoma, N. C. (2015). Working with Modes in Schema Therapy. In *Working with Emotion in Cognitive-Behavioral Therapy: Techniques for Clinical Practice*, 263–287. The Guilford Press.

Stern, D. N. (1985). *The Interpersonal World of the Infant: A View from Psychoanalysis and Developmental Psychology*. Routledge.

Vygotsky, L. S. (1978). *Mind in Society: The Development of Higher Psychological Processes*. Harvard University Press.

Welch, M. G. (1988). *Holding Time*. Simon & Schuster.

Welch, M. G. (2016). Calming Cycle Theory: The Role of Visceral/Autonomic Learning in Early Mother and Infant/Child Behaviour and Development. *Acta Paediatrica, 105*(11), 1266–1274.

Young, J. E., Klosko, J. S. & Weishaar, M. E. (2003). *Schema Therapy: A Practitioner's Guide*. Guilford Press.

Finally, we express our deep appreciation to all the dedicated teams and individuals working tirelessly to promote the well-being of babies and their parents, whose efforts inspire us to continue this important endeavor.
