# Supplementary material for: Integrative mindfulness-based infant parenting program: theoretical foundations and a novel intervention protocol
Source: Front Psychol. 2025 Feb 7;16:1524008. doi: 10.3389/fpsyg.2025.1524008 (PMC11842442; doi:10.3389/fpsyg.2025.1524008)
Supplement: Supplementary file 3 [file Data_Sheet_3.pdf]

Bar-Ilan University

Mindfulness-Based Infant Parenting Group

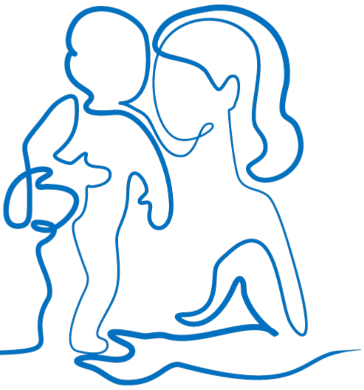

Guidebook for Group Facilitators

Geva Lab
Development, Social Attention and Neuropsychology
https://gevalab.wixsite.com/gevalab

Table of Contents

Introduction **3**

Theoretical Considerations **3**

Developmental stance 3

Modeling mindfulness 4

Being dialectical 5

Vitalizing the learning process with emotions 6

Co-regulation of arousal 6

Working with parental modes 7

Attuning to the zone of proximal development 7

General Guidelines **8**

Minding the learning process 8

Home-work 8

Recap 8

Navigating between meaningful learning and covering the curriculum 9

Communication between guides 9

Every parent counts 9

Setting limits while remaining positive, accepting, and mindful 10

Time is of the essence 10

Full participation in all sessions 11

Setting 11

Communication between sessions 11

Learning with a close partner 12

Intake **13**

Objectives 13

Referrals 13

Setting 13

Intake meeting protocol 13

Protocol **15**

Session I 15

Session II 16

Session III 17

Session IV 18

Session V 19

Session VI 20

Session VII 21

Session VIII 22

References **23**

Introduction

This guidebook is aimed to supply group facilitators with theoretical considerations, general instructions, troubleshooting, and guidelines for conducting the mindfulness-based infant parenting group intervention, along with providing the structure and materials for each session. We will describe the general guidelines, delineate the intake process, and present the protocol for each session. This guidebook is supplied as an online appendix to the published manuscript. Acknowledgments and references to all the theoretical approaches and clinical methods that inspired and influenced the current protocol are provided within the main article.

Theoretical Considerations

It is important that facilitators will be familiarized with the core auxiliary theoretical perspectives of the intervention, including dialectical behavior therapy (DBT), emotion-focused therapy, calming cycle approach, schema therapy, and Vygotsky's learning theory. We encourage facilitators that do not have previous knowledge or experience with one of the approaches to read the following materials before group initiation: for DBT see (Linehan, 1993, 2015); for emotion-focused therapy see (Greenberg, 2017); for the calming cycle approach see (Welch, 1988, 2016); for schema therapy see (Young et al., 2003) or more specifically to mode-work see (Rafaeli et al., 2015); and for Vygotsky's learning theory see (Vygotsky, 1978).

Developmental stance

Grounded on the shared emphasis of DBT and the calming cycle theory, we advocate a dialectical and bi-directional developmental perspective. This means that as facilitators, we focus on the meeting points or the interaction between children and their environment. Accordingly, we present the notion that things never happen only "because of the child" or "only because of the parent"; rather, there is an interplay where additional relational, psychophysiological, social, economic, and cultural aspects also influence the parent, the child, and their relationship. This approach is highly relevant for diminishing judgmental attitudes toward parents. Indeed, the group is specifically designed to elicit change in parents (thereby aiming to positively affect the pathways of child development). Still, we can say to parents, for example, that it is possible that in the future, they could bring their babies to the group where we'll "rectify" their sleep, sociability, or sensory sensitivity and, thereby, modify the parent-child relationship and parents' outcomes (of course, we will apologize, with a sense of humor, that we are still unable to do so, and therefore the burden remains mostly on the parents).

Modeling mindfulness

Mindfulness is the core position we aim to embrace in the group. This involves observing and describing the current experience with an accepting (*i.e.*, non-judgmental) and intentional (*i.e.*, being present) attitude while striving to be effective (*i.e.*, goal-driven).

Being descriptive can be highly challenging, but it is a fundamental position that we aim to foster. This means we try to avoid interpretations and maintain an experience-proximal language that adheres to what our senses capture without drawing conclusions. For example, when parents describe experiences where their child was "angry", we guide them to focus on the overt behavior (*"He started to wave his hands rapidly, while his eyes twitched, and he expressed loud vocalizations"*). It is not that we are against drawing inferences. In units such as *Mindful Responsiveness* and *Minding the Child's Signals*, we acknowledge the importance of translating observations into practical schemes that can effectively guide parental responses. However, we present the distinction between the mindful (*describing*) and the inferring position. And, we value exercising dialectic shifts between these positions (see the *Mindful Responsiveness* module). We advocate the vital role of practicing plain-descriptive, empirical-like language, as it fosters parents' ability to draw more accurate inferences regarding their child. Furthermore, we are required to model the same approach when facilitating group meetings, aiming to acknowledge the distinction between the mindful and the inferring position (*"I saw that your eyebrows rose and your mouth opened when I talked now. I interpreted it like you are saying that you didn't like this idea. Is it accurate?"*). This attitude conveys that translations of observations on others into conjectures on their inner experience (*e.g.*, thoughts, emotions) may not be necessarily precise and that only the person can observe and describe the inner experience most accurately.

Being non-judgmental is an all-encompassing attitude that we maintain right from the intake phase of the group. We do so, first and foremost, by being accepting and non-judgmental as facilitators. Along the group, we encourage parents to observe their own judgmental thoughts and judgmental remarks by other members. However, being non-judgmental does not mean we do not set limits when required. This could be particularly challenging when parents criticize other parents. As facilitators, we must be attuned to judgmental remarks as we aim to establish an accepting and secure milieu. Sometimes, judgmentalism can be very obscure or subtle. For instance, a parent can feel judged when another parent tells what they should have done in a given situation. We can promote the non-judgmental stance by *observing* and *describing* while suggesting that: *"Sometimes when people are telling us what we should have done, it can make us feel sad or judged because it's like it's easy to do. I'm saying that because I saw that you kept quiet after Sara's remark. Is what I'm saying relevant to you in any way?"*. When necessary, we can remind parents of one of the ground principles of the group; that is, we refrain from judging others. We encourage parents to pay close attention and notify the group if they observe judgmental utterances. On the other hand, we acknowledge that everybody can be judgmental at times. We do not seek to "crucify" parents for being judgmental. The agenda is to say that it is natural, but we are all committed to a non-judgmental attitude in this group.

Our approach to mindfulness was influenced by Marsha Linehan's accentuation of the *effectiveness* aspect. We guide parents to be mindful in ways that serve their parental goals. Facilitators utilize the same agenda, for example, when discussing openly conflicting goals (*e.g.*, meaningful learning vs. covering the curriculum) and striving to make effective decisions (*"We see how fertile this discussion is, but we will now move on to the next topic because it's also important that will go through all the units."*). Parents should be guided to be mindful of their own goals and see what is effective for them. This also helps avoid entering into unconstructive quarrels when parents object to assumptions or skills. In such instances, parents are instructed to observe whether this skill is effective for them or are encouraged to experiment with it and observe the outcomes compared to their current preferred mode of operation. This suggests that being goal-driven cannot be the same for all parents, and each parent has their own goals and strategies that can be effective for them (and maybe not for others).

A further extension of the previous point is that we aim to provide a wide variety of channels by which parents can strengthen their "mindful muscle". That is why we concocted an assemblage of various mindfulness-related skills that can be worked through. Some parents will be mostly excited and compelled to practice global mindfulness skills such as mindful breathing or body scanning. In contrast, others will prefer to mostly pick child-related exercises such as mindful interaction. We clearly state that the objective is for each parent to discover, through experimentation, the individually optimal channels for exercising mindfulness in daily life. Although we aim to promote mindful behavior in parenting, we conjecture that the "mindful muscle" can also proliferate through more global exercises, which will eventually affect parental functioning. This suggestion also directs parents to *observe* for themselves what works best for them, thus providing an inclusive, open, and heterogeneous framework.

Being dialectical

The dialectical approach is a way of understanding and solving problems through a process of dialogue in which there is a basic acceptance and curiosity toward the ideas of the other, even in harsh contradictions. One of the basic assumptions of dialectics is that each person has a small share of the truth and, therefore, holds something valid. It may not be the complete truth, but it still is valid. Apart from the basic assumptions section and the indispensable role of dialectics in validation (which were highly influenced by the DBT paradigm), we strive to harness a dialectical approach when working with parents. Sometimes, this is quite hard to achieve, as some ideas will seem peculiar and unfathomable to us. We apply a mindful approach by listening carefully and remembering there is always some sense in what each parent is trying to say (even if sometimes we won't understand it, and we'll need to go on with the curriculum without fully grasping it).

We also aim to enrich parents validation and self-validation abilities. Parents are guided to actively accept themselves – especially by examining ineffective or problematic parental behaviors. We offer a mindfulness-based validation practice involving functional analysis of specific events. When parents use generalizations, we guide them to focus on one particular example and describe in a non-judgmental manner the chain of events in this example ("I was so tired" → "my baby kept on crying" → "I tried to cuddle her, feed her, and caress her for so long

but she just kept on crying"→ "I felt lonesome and desperate" → "I went to my phone and watched videos as if she wasn't there") and then validate ("It's so hard to take care of your child when she doesn't calm and you're tired. Sometimes it's just too much and it's natural to want to take your mind off it"). It may seem counter-intuitive to validate problematic parental behaviors but it is vital for truly integrating this skill and promoting parents' ability to mind and accept themselves.

Vitalizing the learning process with emotions

We seek to inspire curiosity and vitality in the group. We, therefore, invite parents to deal with and work with the tough issues they have as parents and enable genuine and open emotional expression. The experiential aspect of the intervention is nourished in several ways: (1) modeling genuine emotional expression, particularly negative emotions, by group facilitators to legitimize parents' exploration of difficult feelings (Greenberg, 2017; Rafaeli et al., 2011), (2) inviting emotionally charged discourse right from the preliminary stages of the intervention, and (3) specifically inquiring about emotions when exploring parents' dilemmas and home exercises (Greenberg, 2008).

It may be easier to talk about situations in which we were great and did a brilliant job as parents. However, sharing experiences involving adverse parental behaviors or negative emotions may be very hard. Accordingly, facilitators are inclined to demonstrate such personal disclosures. We think that the effect of a facilitator that can share with the group an experience where their parental reactions were adverse for the child while showing mindful expression of emotions and self-acceptance is very transformative and validating. Moreover, such modeling can also carve the way for other parents to talk about themselves more openly and invite the group to discuss sensitive issues.

Co-regulation of arousal

The calming cycle theory emphasizes that the process of regulating arousal is dyadic, and even a small baby is an agent in this process. We do not suggest that parents and babies have the same responsibility, as we believe that parents need to do their best to alleviate distress, effectively address the baby's needs, and achieve calming. However, we suggest that it is important to be mindful of the bi-directional pathways in real-time while acknowledging self and baby. This prism enables parents to recognize cycle patterns with their child and how each agent affects and is being affected in either calming or distressing times.

The calming cycle theory centers parents on vital realms of their relationships with their children – the visceral and bodily realms. It promotes mindful awareness of bodily sensations and tactile experiences. Through this approach, the crucial developmental role of soothing physical contact is expanded and scaffolded by guiding parents to observe and learn what ways are effective for them and their child to calm together.

Working with parental modes

We incorporate the idea that people have several focal voices inside them and can manifest different attitudes or self-positions at different times. In schema therapy, a position of the self (with its thoughts, beliefs, action tendencies, coping attitudes, and feelings) is defined as a mode.

Besides the *Coping Attitudes of Myself and My Parents* module, designed to explore notable parental modes and their relation to childhood experiences with one's own parents, the idea of working with modes is implemented throughout the group. Facilitators will use terms such as *position*, *voice*, *state*, or *mode* to acknowledge the current self that is dealing with the situation. This approach is highly effective for promoting parental mindfulness of the current self-position and for establishing that parents usually shift between positions via the elapsing of time or based on novel experiences.

One example of using this agenda is when parents articulate self-judgmental schemas such as *"It is because I'm a bad parent"* or *"I'm just lazy" or "I had no good reason to be so insensitive to my baby"*. As facilitators, we frame such statements as voices or modes: *"Okay. So, you have this voice that says you're a horrible parent who is just too lazy to take care of her child"*. This allows further mindful exploration: *"Now, maybe you can observe how this voice makes you feel?"* or *"Perhaps you can notice what was the effect of this voice during the interaction?"* (as parents often hold the notion that this inner critic makes them better parents, but through mindful observations may discover that in fact, they become weaker and less effective when such voices arise).

Another position based on schema therapy's notion of reparenting is that modes that inflict self-suffering often stem from unmet needs. To that extent, the role of group facilitators, and possibly also of other parents who collaborate along the way, is to supply novel feedback and more validating responses. *"Well, I'm sure this inner critic you often meet has its rights, but I just listened to how lonely and exhausted you were when you withdrew from your baby, and I say to myself that I would have done much more damage in such circumstances if I were you. Even though it wasn't the best moment, you kept holding your baby in your mind, and 5 minutes later, you were already able to soothe her. So, Mr. Critic, I think we shall give Rachel some break"*. When passing such comments, facilitators should aim to be *descriptive*, use mindful appraisals, reflect concise descriptions of the chain-of-events, and refer, compliment, or empathize with a specific behavior (rather than merely using general compliments such as *"You're a great parent"*). The idea is to try to help parents make sense of a specific behavior in a way that realizes the nexus between their emotions and needs, the child's responses, and their behavior, while supplying positive regard.

Attuning to the zone of proximal development

Vygotsky suggested that learning transpires when a more knowledgeable agent scaffolds the interaction in ways that enable the other to grasp something new that the other can absorb in a given moment. We suggest that effective Vygotskyan parenting requires mindfulness, such that parents need first to grasp the child's current ability and state and then direct their parenting behaviors within the zone where meaningful learning could

materialize. We adopt the same attitude as guides. Facilitators mustn't try to look smart for the sake of appearance; facilitators should talk in a way that is clear and helpful for the participating parents. We try to use plain language, not assuming that parents know professional terms and words beforehand. We also raise awareness of the learning process within the groups by often asking whether our explanation of a specific skill or idea was clear or whether a specific practice was effective for them.

General Guidelines

Minding the learning process

Throughout the group we work with written materials from the handbook. We invite parents to read aloud materials. This is not only for technical reasons. We guide parents to pay attention to the learning process, suggesting that for some, reading aloud can be effective (for staying active or attentive, for understanding, etc.), while for others, listening or observing the text visually is more effective. We want parents to be aware of what helps them learn more proficiently. When working with texts, we usually start by asking: *"Is there someone who wants to read for us? Or thinks it can be effective for them now?"*.

Home-work

We encourage parents to practice. We convey the message that training is fundamental for eliciting change and actually taking in something new and helpful from the group. However, we think the most important thing is that each parent will find their own way to exercise. We guide parents in finding their own effective approach to assimilating the skills at home. Some parents work best with structured chores, but others exercise the skills daily without filling out even one handout. We must acknowledge that this can also be effective and that there is no one right way to train. Nevertheless, we want to reinforce disciplined home-work. Therefore, when we go through the home-work at the beginning of each session (usually after the recap), we will first invite parents who completed the home-work as instructed and subsequently move on to hear other parents (of course, we shouldn't be rigid about it; if a parent that usually doesn't speak wants to discuss working with a specific skill at home even if they did not fill out the handout, it will be wise to make an exception).

Recap

From the fourth session, we begin each session with a short recap of the previous session. The recap could often be used to bring up to speed parents who missed the last session. In the recap, we invite one of the parents to briefly summarize the contents of the previous session. We think this aids parents in making the theories and skills their own, using their own language. We can ask for a volunteer to conduct the recap of the next session at the end of each session.

Navigating between meaningful learning and covering the curriculum

One of the most challenging tasks for facilitators is to navigate between the goal of going through the entire curriculum and the need to inspire meaningful learning experiences. There is no easy answer to how this should be managed. It is not good enough if the entire curriculum is covered. We want to enable spirited discussions and reach each participant. To that extent, facilitators can have the liberty to slightly modify sessions. However, it is important that facilitators also keep in mind the importance of covering the curriculum and not leaving out essential modules. Ultimately, the approach should be dialectic: the interplay between the two goals should be weighed and discussed between facilitators throughout the group.

Communication between facilitators

Facilitators should strive to establish an effective, open, and positive rapport, as it resonates in the group's atmosphere. It is best if facilitators have a preexisting effective professional relationship. But it is not a prerequisite. If this is the first collaboration between facilitators, they should meet and familiarize themselves while openly discussing their preferences and sensitivities.

Before each session, facilitators should decide who the dominant speaker of each section of the session is. The dominant facilitator guides the section, but the other facilitator can also contribute. Establishing non-symmetrical deployment of roles is fine as long as they are established in dialogue and mutual acceptance. For example, if one facilitator feels less comfortable orchestrating mindfulness exercises, there is no problem that the other facilitator will be in charge of these exercises throughout the group. The most essential factor is that facilitators work well together, communicate openly, and conduct the group effectively. The fact that facilitators are not the same is also fine; personal differences can be used as a model for parents and can be openly discussed.

At the end of each session, after the parents leave, facilitators should conduct a 15-minute talk about the session, addressing issues such as the participation of all participants, specific dilemmas, covering the curriculum, or other issues that arose.

Every parent counts

Our essential attitude is that each and every participant is important, and we make an effort to provide a meaningful learning experience for each one. This suggests that facilitators should be attentive to the more silent or "drifting" parents. There are some exercises where each parent is invited to speak. However, in many others, participation is not actively called upon by each one. After the first three meetings, there are usually more dominant and less visible speakers. We should acknowledge that talking is not equivalent to learning and that there are many ways to learn. Facilitators should state the objective of enabling each parent to have a meaningful learning experience. It can be helpful to deliberately invite specific – more "silent" – parents to participate. Facilitators can first describe what they observe (*"Daniel, I wanted to see if you'd like to start this discussion, as*

I've noticed that you haven't talked in the recent meetings"), be non-judgmental and accepting (*"Will it be effective for you to talk"*; or *"I don't think that talking means learning, but it was important for me to hear you out, and see whether the group is effective for you, or maybe we're missing you here"*), and raise self-awareness (*"Some people learn best by listening, others by talking, some by practicing"*). We want each parent to feel they are important. However, we should be mindful when approaching such parents. If facilitators observe reactions that may indicate discomfort, they should acknowledge them (*"I'm sorry if the way I approached you was not comfortable for you now"*; if the parent verifies such an assumption, the facilitator can take responsibility and say they're sorry for pushing in a non-effective way, and thank the parent for helping them understand better).

Setting limits while remaining positive, accepting, and mindful

Some parents tend to speak more often and sometimes in ways that shift the discussion from the primary goals of the session or don't leave enough room for others. Facilitators should work with such parents with compassion while remaining focused on the objectives of the intervention. We aim to orchestrate sessions in a way that enables us to cover the curriculum and stay oriented on the skills and their implementation. There are many reasons that specific parents will show such a tendency; it could be related to increased impulsivity, a need to be heard and appreciated, anxiety regarding urging issues with the child, trauma, or others. All along the way, facilitators should implement a non-judgmental attitude toward parents and try to understand the underlying needs that drive each one. This approach can help when we set limits. We should clearly state that we want to be effective and cover all the curriculum, and therefore, we have to stop some discussions, even if they can be highly relevant and helpful. Facilitators should also state the objective of making room for all parents to speak and, accordingly, choose parents who haven't talked before parents who are frequently eager to speak. However, when doing so, we should acknowledge the positive aspects of each parent's participation (including the more talkative ones). For example, facilitators can speak about the positive impact of a parent who often tends to raise criticism and skepticism regarding the skills, stating how it helps clarify the ideas and consider their practical utility. Facilitators can also state that it is natural that some parents tend to speak more in groups, some need more time to feel comfortable to share, and some prefer to listen.

Time is of the essence

Sessions start on time! From the intake, we aim to establish an agreement with each participant that arriving on time is imperative. Facilitators invite participants to arrive 15 minutes before the beginning of each meeting. We want to make this pre-meeting period appealing. This is a time to make coffee together or eat some refreshments. Facilitators should be available during this time, as it allows informal conversations that help connect and get together. Sometimes, it can even be a place to discuss some issues parents had during the week while practicing the skills. Nonetheless, we start the session on time, even if some parents are late. We ask parents to inform us beforehand if they will be late so we can be better prepared. Of course, we always maintain a non-judgmental

attitude toward parents, but we state that the group's effectiveness depends on starting on time, and therefore, we make an effort to make it happen.

Full participation in all sessions

Agreeing to attend all sessions is a prerequisite for participation. We acknowledge that parents may have some events or situations that prevent them from attending a specific session. We ask parents to notify facilitators as soon as possible. We bring up this issue during the intake. It enables parents to think and prepare better for the group and see whether it can be feasible or even possible for them to participate during this specific period of their life. To promote adherence, we set the rule that missing more than two sessions means termination of participation in this particular group. We tell parents that these rules aim to promote effectiveness and enable parents to consider beforehand whether participating in the group can suit them now.

Setting

The group is conducted in a convenient room with sufficient space. Sitting is arranged in a circle (using chairs, sofas, couches, or other comfortable seats), while group facilitators sit beside each other within the circle. The room needs to include a TV screen or projector that could be used to screen the video materials. There should be enough pens or pencils for all participants, and extra handbooks for parents who forgot their personal handbook at home. The room should be neatly prepared 15 minutes before the start of each session. We think that having coffee and refreshments available when parents arrive and during breaks is important. Eating and making coffee together first soothes the basic need for eating and drinking, and even more so, it enables time to familiarize, connect, and even have informal discussions regarding relevant parental dilemmas.

Communication between sessions

Generally, facilitators are not required to communicate with parents between sessions. However, there are instances where communication between sessions is called upon. First, we instruct parents to inform us beforehand if they can't attend a specific meeting or will be late. Further, sometimes, we might encounter a parent who seems distressed, angry, quiet, or anxious. More specifically, sometimes, we can observe adverse reactions of parents to something we said during the session. Although we must keep in mind and state that the relationship is not supposed to be equivalent to what is proposed in one-on-one psychotherapy, we try to resolve such issues or convey to the parent that we noticed something happened. We do that because each parent is important to us, and we want to enable a meaningful learning experience for each one. In such instances, we actively call the parent and act as caring fellow human beings. If we think the change in the parent's behavior was related to something we've said, we can describe what we saw and our inference that we might have caused discomfort. Then, we basically stay mindful and listen. Nevertheless, facilitators mustn't assume the role of personal therapists. If major issues arise (*e.g.*, acute distress, suicidal ideation, major psychiatric symptoms, etc.), we

should refer parents for treatment, stating the limitations of the group. However, we should also say that we believe and hope the skills we learn in the group will help the parent with these issues, even though they may not be a sufficient solution.

Learning with a close partner

We encourage parents to participate in the group along with a partner. A partner can be the additional parent (for couples raising a child together) or another meaningful other that plays a role in raising the child (*e.g.*, grandparent, close relative). In the intake, we strongly encourage parents to come along with the partner, even if there is a very slim likelihood that the partner will be able to participate in the group. We state the rationale for participating together, involving the merits of going through a meaningful process with a significant other. We make an effort to consider together whether there is a possibility for enabling partners to come along. However, we mustn't be too rigid about it. Some single parents don't have a significant other that assumes a meaningful role in raising the child. In such instances, it is very invalidating to "push" for finding a partner for the group (it is somewhat more suiting to appraise the parent for the devotion and commitment to the child). In other cases, it is simply not feasible for both partners to enroll (*e.g.*, due to lack of motivation of one of the partners, work obligations, etc.). Facilitators must validate and acknowledge that parents are doing their best and reinforce their openness, which enables them to weigh the feasibility of participation wisely.

Intake

Objectives

The main objectives of the intake phase are as follows:

1) To establish whether effective participation (*i.e.*, attending all sessions on time) is feasible.
2) For parents who wish and can participate, establish a "contract" regarding the primary requisites (*i.e.*, attending all sessions on time).
3) To supply brief psychoeducation regarding mindfulness, mindful parenting, and the group's setting and objectives.
4) To establish an initial positive rapport between facilitators and parents.
5) To enable a preliminary experience of what the group offers involving mindfulness skills.

Referrals

Whether the referral to the group was by the parents, through an ad, via a recommendation by friends, therapists, or a clinic, the intake meeting is mandatory and involves the same process, following the abovementioned objectives.

Setting

Both facilitators should be present in the intake meeting. It is recommended that the meeting be held face-to-face. However, conducting it online as a video meeting is also possible. The allotted time for the meeting is approximately 30 to 40 minutes.

Intake meeting protocol

The meeting starts with a brief acquaintanceship. Facilitators state their names and professional qualifications and ask the parents for their names, occupations, and the name of the baby that brought them to the meeting.

We then ask parents what interested them in the group or why they decided to seek this group. This question already prompts parents to be mindful of their objectives and drives while also enabling them to assess whether their goals align with what the group offers. We state that the main objective of the intake meeting is to consider together whether the group could be effective for the parents. We want to hear each parent and suggest that both parents may share the same goals, but sometimes, it is not the case, and in any event, it is important for us to hear each one. We listen carefully and think about the goals in the context of the group.

Subsequently, we ask parents whether they have prior experience with mindfulness or mindful parenting. If so, we ask about their experience. For parents with prior experience, we suggest that the following briefing might

be familiar to them, but it is important for us to state what the group is about. We then articulate a short definition of mindfulness and discuss the means and merits of implementing mindfulness in parenting (*i.e.*, mindful parenting). We elaborate the definition with at least one tangible example that shows how mindfulness can contribute to parents raising babies (*e.g.*, for scaffolding language acquisition when parents articulate a novel word in nexus to the child's investigation, in enabling parents to be better aware and take better care of their own needs and feelings). We state that mindfulness has been shown to aid people in promoting well-being, and, more specifically, parental functioning and parents' well-being. Following this briefing, we ask parents what they think and collaboratively assess whether such skills are congruent with the objectives of each parent. We also ask if they have any questions regarding the group or mindfulness skills.

We then state the basic setting and requisites for participation. We state the dates and times of the meetings and clearly state that participation involves a commitment to attend all sessions on time. We say it is important to state this beforehand because we think partial attendance is ineffective for the parents and the entire group. Therefore, we aim to substantiate whether participation in the current group is feasible. Facilitators shouldn't be afraid if parents realize that they can't participate. Facilitators remain accepting and non-judgmental throughout the meeting, appraising parents' ability to mind their needs, goals, and life circumstances even if the conclusion is that participation is currently not feasible. However, we state the positive aspects parents can gain from the group. If there is a dilemma, we strive to weigh all factors together and see whether a viable solution enables parents to participate. We state that parents may encounter a specific problem in arriving to one of the sessions (for example, because of sickness or family event), and, here, we can show positive regard by suggesting that the mere willingness to consider partaking in such an endeavor when raising a baby, managing a family and career (etc.) is admirable and cannot be taken for granted. We suggest that missing one session could be contained. Still, we believe that missing more than two doesn't enable an effective learning process and, therefore (and also for fostering adherence), will result in the termination of participation in this specific group.

We then ask parents if they have any other questions or things they want to share or state. In summary, we want to link parents' reasons for considering the group and the possible merits they can gain from participation. We greet parents personally and thank them for the opportunity to meet (whether they decide to participate or not). If they wish to participate and concur with the requisites, we share our wish to meet and work together in the group. Sometimes, parents have conflicting needs that call for further consideration. We mustn't push such parents to decide immediately. We want to raise awareness, and aid them to decide wisely. This could be done by setting a date for their decision (usually a couple of days following the meeting) and inviting them to call or text us if they have any other questions.

Protocol

SESSION I

Min	Unit/Topic	P.	Description
30	Getting Together Circle	-	Along with introducing names, each parent is invited to share a specific "cute" or special moment with the child. The idea is to ask parents to be mindful right from the beginning. We specifically guide parents not to say something general but to choose a specific moment they remember. An interaction, something cute they noticed the child did, a shared experience. Even if it is recurring, we guide parents in choosing and describing a specific event.
5	Introduction	2	We go through this page very briefly. We usually don't read the entire intro at the upper half of the page. Try to summarize it in a few sentences. Then, we read the Group Goals sub-section together.
25	The Scaffolding-Feedback Developmental Model	3	We read together the model description in the upper section of the page. We read with pauses, leaving room for questions and discussion. We then move on to the lower section. Here, we can give some examples to acknowledge the dynamic dimension, such that the same parent may form different relationships with each of their children. For instance, if a parent went through a divorce right before the birth of the second child and was sad, lonely, and fatigued, it may affect the child and the relationship they form. Alternatively, a child with a very difficult temperament (hard to soothe, not sleeping well, etc.) may destabilize parental sleep or mood, thus affecting parental functioning and the relationship. We ask parents for their own examples.
10	Break		
40	Assumptions	4	We suggest that these are ground assumptions we encourage parents to adopt. We want to designate sufficient time for the assumptions page. They set the ground for focal positions, such as being non-judgmental and validating. We read each point carefully and then invite parents to say their thoughts. Usually, the first assumption stirs various reactions. We want to hear them out. Many parents might say that they don't do their best, are just lazy, or that their partner is. We suggest that the idea that parents do their best does not mean that their reactions are effective. More than that, they might even be harmful at times. Nonetheless, there are reasons for harmful parental behaviors, and we suggest that embracing this assumption promotes the likelihood of understanding ourselves and finding better solutions. When there is a tough opposition to this idea, we can invite parents to keep it in mind and see whether the assumption can be effective for them. The dialectic approach is accentuated in the shift to the second assumption. We want to emphasize it. On the one hand, we do our best, while on the other, we must strive to do better and improve.
8	Principles for Productive Work in the Group	5	We briefly go through the text.
2	Preparing for the Next Session	-	We discuss the purpose of the next session, in which we want to make room for meaningful experiences from the early phases of the child's life to be processed together. We explain that the next session will be without a break to sustain an enabling flow. Therefore, we encourage parents to come early and enjoy the coffee and refreshments before we start.

SESSION II

Min	Unit/Topic	P.	Description
120	Formative Experiences from the Ealy Stages of Life	6	We start by reading the upper section of the page together. We then deploy approximately 15 minutes to allow parents to write their experiences on the page. Facilitators can write about their own experiences along with parents. We then invite parents to share. We emphasize that we want to allow each parent to share and that we will try to avoid comments and listen carefully with our minds and hearts. Suppose we notice stories that include self-judgmental attitudes of parents regarding their behavior or reactions. In such cases, we will mark them to ourselves. After the parent finishes talking, we will offer validation and reframing of the situation, accentuating how the parent did the best she/he could in the given circumstances. Facilitators should allow themselves to be emotionally engaged, thank each parent who shares, and, when dealing with adverse or traumatic experiences, acknowledge how hard, sad, frightening, or painful the experience was. If there is no description of emotions, facilitators can gently ask what parents felt during the experience. However, we remain mindful and want to foster parents' awareness of their limits and the pace of processing that suits them. We avoid pressuring parents to share and acknowledge that each one knows the right pace for her/him. If there is sufficient time and facilitators have relevant experience, they can also share it with the group, as we suggest that it promotes bonding processes, safety, and partnership. At the end of the sharing circle, we read the lower section of the page, acknowledging parents' courage and openness for sharing, which allowed us to learn, empathize, and connect.

SESSION III

Min	Unit/Topic	P.	Description
10	Body-scanning	-	We begin the session with a body-scanning exercise administered by one of the group facilitators. Facilitators can use exercises such as the one articulated by Jon Kabat-Zinn from *Full Catastrophe Living* on pages 92–93 (Kabat-Zinn, 1990). The exercise needs to be modified for a sitting position. Following the exercise, we conduct a short inquiry, inviting parents to share something they noticed, difficulties they had during the exercise, some discomfort they may have noted, etc.
20	What is Mindfulness?	7	We start by stating that we are now entering the realm of mindfulness and that our initial port of entry will be through global or more general mindfulness skills and the broader definition of mindfulness. And subsequently, we will synthesize these skills into parenting. We read the definition at the top together. We specifically focus on the aspect of the present moment. Accentuating that mindfulness is about bringing awareness to the here-and-now. We then invite one of the parents to read the rest of the page. We leave room for questions and discussions.
20	Mindlessness	8	After reading the instructions, we take 5 minutes to fill out the page and allow some parents to share their familiar experiences of mindlessness. We also convey the notion that being mindless is not always "bad", as it allows us to free our resources, as sometimes our mind needs some rest.<br>Home-work: Parents are instructed to choose a behavior they often do when mindless, and during the following week, on three separate occasions, do it intentionally while focusing on this behavior only.
5	Mindfulness Video	-	Watch together a video about mindfulness. One possibility is the video *Why Mindfulness is a Superpower* by www.happify.com (Credits: narration – Dan Harris; animation – Katy Davis; animation assistant – Kim Alexander).
10	Break		
20	Core Mindfulness Skills: Observing and Describing	9	We explain that we will now go through the core mindfulness skills. We briefly name all five and then say that today, we will go through the first two skills – *observing* and *describing* – together and then exercise them. We read the paragraph on *Observing* and emphasize the importance of focusing on one thing at a time. We can use the metaphor of a lens that is purposefully oriented toward a specific target/object. We also accentuate the centrality of present-centered observation, that is, focusing on what we experience now. We then move on to read the *Describing* paragraph. After reading, we emphasize that it is not necessarily easy to do and demonstrate it by asking one of the parents to describe an object in the room or do it ourselves without using interpretive language, addressing only physical or spatial manifestations such as size, color, thickness, orientation, placing, material, etc.
20	Mindful Eating Exercise	-	The classical exercise by Jon Kabat-Zinn (Kabat-Zinn, 1990) involves raisins. We offer parents several options (can be nuts, raisins, cookies, etc.). Each parent is guided to choose one. We then use the following Instructions: *"Now, let's first observe the food; what do we see? We can describe the visual appearance or write it down. Now, we can bring it closer to our nose and smell it. What odors do we notice? Let's move on to observe how the food feels in our hands. We can gently touch it. Feel its textures. Is it firm? Soft? Do small bits crumble when we touch it? We can place the food in our mouth, but slowly. No chewing yet. Just observe the food in the mouth – the weight, texture, other sensations that the food evokes. Try to describe them for yourself. You can also write them down. Now, we'll try to eat slowly, chewing 10 or even 20 times. Pay attention to the tastes. Aromas. Changes in the texture while eating. And decide for yourself when to swallow. Do it deliberately. Focus on this process, the food traveling along the food pipe. Can you track it? Has it reached the stomach? Observe your related feelings. Are there feelings of fullness and satiety? Are you hungry? Let's try to reflect on the current experience of mindful eating. Were there things that surprised you? Did you notice anything interesting? Please write for yourself some of these reflections."* We follow the exercise with a short inquiry, inviting parents to share their experiences, sensations they noticed, or reflections.
5	Home-work: Mindfulness in Daily Routine	10	We read the instructions on the upper section of the page together. We guide parents in choosing one activity and doing it mindfully once during the coming week, following the instructions on the page, and filling it out after the exercise.

SESSION IV

Min	Unit/Topic	P.	Description
5	Recap	-	We invite one of the parents to summarize the primary skills/topics of the previous session.
5	Mindful Breathing	-	Facilitators can use the exercise supplied by Decker and his colleagues (Decker et al., 2019) in Appendix A in their manuscript. We invite parents to try to exercise mindful breathing at home.
10	Home-work	-	We invite 2–3 parents to share their experience with the home-work and conduct a brief inquiry while focusing on whether the exercise was effective for them, were there related difficulties, etc.
30	Core Mindfulness Skills: Being Present, Non-judgmentalism, and Goal-driven	9	We continue learning the core mindfulness skills. We read each skill carefully. After one of the parents or facilitators finishes reading a skill, we pause and invite questions and discussion. We go through the *Being Present*, *Non-judgmentalism*, and *Goal-driven* paragraphs. We integrate the skills and, for example, suggest that to apply a non-judgmental stance, it is necessary to be able to observe and describe the current experience as it is.
10	Break		
30	Judgmental vs. Non-Judgmental Stance Exercise	11	We read the description together and then deploy approximately 8 minutes to fill the page. We then conduct a brief discussion, inviting parents to share. Subsequently, we go through the home exercise at the bottom of the page.<br>Home-work: We read the first paragraph together and then the instructions below.
20	Mindful Moments	12	We go through the page together. We read the descriptive section at the top and then the instructions for *Taking a Mindful Moment*. We emphasize that implementing mindfulness does not necessarily mean stopping everything altogether. It can also be used in brief moments during our daily routine and when nurturing the child.<br>Home-work: We read the instructions together and guide parents to exercise during the following week and write down their experience in the bottom section of the page.
10	Selective Attention Video	-	We watch together the video from the pioneering study by Simons and Chabris (1999). We say some parents may be familiar with the video and ask that they also watch along, refraining from comments. The video can be found on Viscog Productions (www.viscog.com). Following the video, we ask parents who noticed the gorilla. We can use this experience to accentuate several important notions: (1) That our goals can navigate our attention to some aspects of reality while blind us from others; (2) That sometimes we might be stuck in a tunnel vision and that it is important at times, to be open and observe the situations from an open and curious approach, especially when we are with our child (as sometimes we can be consumed by a goal we have – such as feeding the child – that we might miss something that is going on with him/her); (3) The idea of autopilot: suggesting that we might miss important signs when we work on autopilot. We don't need to address all of these ideas, but we can use one or some of them during the discussion with the parents.

SESSION V

Min	Unit/Topic	P.	Description
5	Recap	-	
10	Sensory Awareness Exercise	-	Facilitators can select a sensory awareness exercise of their choosing; we recommend administering an auditory awareness exercise. Here are some options: (1) *Sitting with Sound* exercise in *Full Catastrophe Living* page 72 (Kabat-Zinn, 1990); (2) *Who is Listening?* exercise in *Practicing Mindfulness: 75 Essential Meditations to Reduce Stress, Improve Mental Health, and Find Peace in the Everyday,* pages 10–11 (Sockolov, 2018). We conduct a short inquiry following the exercise, inviting parents to share insights and describe their experiences. We ask parents to try to exercise sensory awareness at home.
10	Home-work	-	We invite 2–3 parents to share their experience with the home-work and conduct a brief inquiry. Parents can share their experience with the *Non-judgmental Stance* or *Mindful Moments* exercise.
30	Mindful Attention with the Child	13	We start by stating that we have finished going through more global mindfulness skills and move on to focusing on the group's primary goal, namely, using mindfulness in parenting. We invite one of the parents to read the paragraph at the top of the page. We leave some room for discussion and then move on to the meeting video exercise. We watch together a still-face-like video. An intriguing video that could be used for this exercise is the *Still face experiment* video by Lise-Lotte Austad. This video depicts an ecological still-face reminiscent experience in which the typical behavior of a parent using a smartphone during an interaction with the baby affects the baby's state. The video can be accessed at: https://www.youtube.com/watch?v=bOR7jId8wYk. Before viewing the selected still-face video, we read the instructions and allow parents a few minutes to fill in their impressions regarding the baby's reactions. Using this video serves two goals. First, exercising mindful attention to the child. Second, there is an accentuation of the imperative role of mindful parental attention to child development and the adverse impact that parental inattention to the child might have.<br>Home-work: We read the instructions together and guide parents to exercise observing and describing the child during the coming week.
10	Break		
30	Mindful Responsiveness	14	Many parents tackle the notion of mindfulness, suggesting that merely deliberately observing and describing without being judgmental is not enough, as they have to actually respond to the child's needs. Until this point, the main response to this notion is related to the *Goal-driven* aspect of mindfulness that we articulated. However, in this module, we aim to supply a broader answer to this issue, as our main agenda is not to sell mindfulness for its own sake but rather to suggest that mindfulness can promote parental functioning, parents' well-being, positive bonding processes, and child outcomes. Here, the dialectical position is at the core. We suggest that being able to switch between the mindful, inferring, and behaving parental modes is fundamental for responding wisely and effectively to the child. This also includes building upon global developmental knowledge and specific knowledge parents gathered about their children. We read the page together and pause following each point to enable discussion while accentuating the abovementioned points.
5	Minding the Child's Signals	15	Home-work: We read only the first paragraph of the instructions and guide parents to exercise and fill in the page.
20	Mindful Interaction	16	We read together the first paragraph. We emphasize that to foster positive parent-child bonding processes and to promote secure attachment patterns, parents need to be present during interactions and that children require times of holistic and deliberate presence of their parents. We also accentuate that this is not the only thing parents need to do in life. Parents must also care for themselves, feel loved and respected, eat, sleep, and more. We want to emphasize this to reduce judgmental attitudes toward parents. We do not believe that parents can or need to supply mindful attention every second of the day. We want parents to hear that because we think that sometimes taking care of oneself is the most effective thing a parent can do to also take better care of the child. Nonetheless, we invite parents to promote the daily implementation of this exercise to the best of their ability.<br>Home-work: We read the *Mindful Interaction Exercise* box and then the instructions below, and we guide parents to conduct this exercise at least once this week and fill in the page.

SESSION VI

Min	Unit/Topic	P.	Description
5	Recap	-	
10	Home-work	-	We invite 2–3 parents to share their experience with the home-work and conduct a brief inquiry. Parents can share their experiences through an exercise of their choice.
15	Experience-Proximal Elaborations	17	We read together the first paragraph and use the figure to facilitate parents' understanding of the conceptualization. Subsequently, we read the definition of *Experience Proximal-Elaborations* (blue shading). Regarding the description of the types of elaborations based on Daniel Stern (Stern, 1985), we do not go through it all but merely summarize it by suggesting that there can be many forms of elaborations: elaborations that add another shape (or sensory modality) to the child's experience (*e.g.*, adding vocalizations of a rattling engine when the child is driving a toy truck), change the intensity (*e.g.*, repeating the baby's vocalizations but more subtly), change in rhythm (*e.g.*, repeating the child's movement but more slowly), or combination of several types. We also emphasize that through elaborations, parents can scaffold their child's language acquisition when uttering a word at a precise time when they are in a tight nexus to the child's current exploration. We emphasize the crucial role of mindfulness in employing effective elaborations, as in order to be proximal, a parent must first grasp the child's current experience.
35	Self-Validation for Parents	18	We go through the page while enabling debate. We stress the importance of validation in our perspective for reducing self-judgmentalism and promoting the mindful position. We suggest that parents also need others to validate their needs and emotions and acknowledge the hardships they might endure when their environment is invalidating. Some parents may suggest that some behaviors are unworthy or cannot be validated. We suggest that we offer validation not to make parents feel good about themselves (although that is also fine) but to promote better parental functioning. We guide parents to observe whether validation can be effective for them and how they usually respond when invalidated. Some parents hold high regard for self-criticism and may advocate that self-criticism allows them to better themselves. Again, we do not fight with this idea. We state that this might be true for some people and call for each to explore whether validation and self-validation can be effective for them. After reading the entire page, we invite parents to share experiences of self-invalidation or being invalidated by others and exercise together, validating the parents' responses, feelings, thoughts, and attitudes. Even in adverse behaviors, we try to trace the functional purpose of the behavior – even though it may not have been effective. When exploring parents' examples, we guide them to choose a specific event and describe its chain-of-events, including behaviors, thoughts, emotions, external factors, and the reactions of the other. We then invite parents to try to validate others and inquire each parent regarding the emotional response that being validated has elicited. Usually, parents will observe and describe a positive or soothing emotion. However, at times, this may not be the case. Sometimes, we encounter a rigid inner critic suggesting that the parent shouldn't listen to the validating response that she/he doesn't deserve. We can respond using a mode-oriented and dialogic approach: *"We see you have this inner critic that thinks you responded that way because you are bad. Well, we think differently. We think you did your best and deserve compassion for the tough situation you were in."*
10	Break		
25	What Parents Feel is Important	19	We read along the page, pausing after each section to enable questions and discussion. Parents may find this skill counter-intuitive. We remind parents that we want to foster viable conduits for calming and staying close within the parent-child relationship. Again, we use the mindful stance, instructing parents to practice and observe whether this approach can be effective for them.
20	Parent-Child Mutual Regulation	20	We connect this page to our basic developmental theory, which accentuates the interrelations between child and parent while focusing on the role of joint regulation of arousal (especially in moments of distress). We read the first paragraph and then make the idea more straightforward using the figure. Home-work: We read the instructions below and guide parents to observe and write down a pattern of mutual co-regulation (or co-dysregulation) they will experience with their child during the following week.

SESSION VII

Min	Unit/Topic	P.	Description
5	Recap	-	
10	Home-work	-	We invite 2–3 parents to share their experience with the home-work and conduct a brief inquiry. Parents can share their experience with the *Parent-Child Co-Regulation* exercise or their experiences with validation, self-validation, or invalidation.
15	Mindful Coping with Distress	21	We read the upper section of the page together.<br>Home-work: The task involving describing an interpersonal incident of distress with the child is given as a home chore.
15	Interpersonal Distress	22	We first go through the scheme at the top of the page, with the arrows leading from an event to the solution of the distress. Our suggested scheme emphasizes that even though some reactions might be truly adverse, there are reasons they occur. There is a natural tendency to try and alleviate distress, and every response to distress could be conceived as an effort to deal with it. We then read the box regarding the typical characteristics of distressing situations. Subsequently, we read together the section on mindful coping with distress and introduce the *Distressometer* as a graphical tool to help parents be more mindful of their current inner state. Here, we already suggest the notion that understanding that we are in distress is the most essential factor in determining whether we manage to deal with the distress mindfully.
5	Validation Video	-	We watch together a video about validation. The video aims to scaffold the assimilation of this skill and reflect its essential role in parental functioning and well-being. If possible, an excellent option is viewing three segments from episode 17, season 2, of the TV show *Modern Family*, titled *Two Monkeys and a Panda*, (directed by Beth McCarthy-Miller, written by Christopher Lloyd, Steven Levitan, Carol Leifer and Elaine K; first segment starts at 08:20 and ends at 09:27; second segment start at 12:30 and ends at 13:55; third segment starts at 15:30 and ends at 17:41) – we think these segments are magnificent as they are funny, and summarize the idea of validation brilliantly while considering familial relationship including parents and children. If this video is unavailable, another option is to view the video *Brené Brown on Empathy* (Credits: voice – Brené Brown; animation – Katy Davis; production and editing – Al Francis-Sears and Abi Stephenson). Although this video uses the word empathy and not validation, the explanation is consistent with the notion of validation we wish to convey (if viewing this option, we can tell parents that the idea of empathy in this video is highly similar to the concept of validation we learned). The video on empathy was published by the Royal Society for the Encouragement of Arts, Manufactures, and Commerce (the RSA; https://www.thersa.org) and can be accessed at: https://www.thersa.org/video/shorts/2013/12/brene-brown-on-empathy.
10	Break		
45	Coping Attitudes of Myself and My Parents	23	We read the first paragraph and then deploy 10 minutes to let parents complete the page. We then invite parents to share. We try to make room for all parents to contribute to this talk. We emphasize the notion that sometimes there are modes that get "stuck" with us, even though we don't necessarily like them, due to meaningful experiences we had as children. We try to raise awareness of the predominant parental modes and promote parents' ability to observe their current mode, as well as possible alternative modes.
15	Regulating Distress Together	24<br>25	We read the page together. We emphasize that each one can build their own arsenal of skills for regulating distress that is effective for them. We also encourage parents to find applicable ways to regulate stress together with the child, emphasizing the importance of interpersonal means for regulating distress.<br>Home-work: We briefly go through the instructions of the upper and lower sections of page 25, and guide parents to experiment with skills for regulating distress, and observe the outcomes to develop a personal basket of effective ways to alleviate distress with the child.

SESSION VIII

Min	Unit/Topic	P.	Description
5	Mindfulness Exercise	-	We start the session with a brief mindfulness exercise. We can repeat one of the exercises we conducted in previous sessions (*e.g.*, mindful breathing, sensory awareness, body-scanning).
5	Recap	-	
10	Home-work	-	We invite 2–3 parents to share their experience with the home-work and conduct a brief inquiry. Parents can share their experience with either one of the exercises involving interpersonal distress tolerance skills.
30	Embracing Our Pain	26	We read the page together. When explaining the notion of *Embracing Our Pain,* we can also use the metaphor of riptide, in which, to survive, it is advised to let yourself be drawn with the current inwards until you reach the place where you are thrown out of the current. Alternatively, when you fight the riptide, you exhaust yourself, thereby increasing the risk of drowning. Accordingly, in this position, we accept the pain and let it sweep us entirely until, eventually, it will pass. The idea is that sometimes fighting the pain makes it stronger, and acceptance allows us to move on. We acknowledge the dialectic tension between the previous skills aimed to alleviate distress by changing the current experience and the embracing position that mindfully accepts the present experience. We suggest that parents should be able to use both approaches, which can be effective in different situations.
10	Break		
60	Concluding Talk	27	We direct parents to observe the word board (it is helpful also to display it on a screen). We guide parents to observe the words and consider two questions that will help us summarize and appraise the group, considering (1) What was the primary skill they have implemented in their daily parenting, and (2) What skill they wish to improve in the following year. We deploy 7 minutes to write down the responses. The semi-structured approach focuses the discussion on the skills and their applicable relevancy for each parent. In this talk, we listen to each of the participants. Subsequently, each group facilitator offers their own summation of the group, striving to find their meaningful and enlightening experiences during this specific group. The concluding talk by facilitators should relate to specific experiences in a mindful (*i.e.*, descriptive) way and acknowledge the positive aspects of this specific group. We want to send the participants home with the positive emotional feedback they deserve.

References

Decker, J. T., Brown, J. L. C., Ashley, W., & Lipscomb, A. E. (2019). Mindfulness, meditation, and breathing exercises: reduced anxiety for clients and self-care for social work interns. *Social Work with Groups, 42*(4), 308–322.

Greenberg, L. S. (2008). Emotion and cognition in psychotherapy: The transforming power of affect. *Canadian Psychology / Psychologie Canadienne, 49*(1), 49–59.

Greenberg, L. S. (2017). *Emotion-Focused Therapy: Revised Edition.* American Psychological Association.

Kabat-Zinn, J. (1990). *Full catastrophe living, revised edition: how to cope with stress, pain and illness using mindfulness meditation.* Delacorte Press.

Linehan, M. M. (1993). *Cognitive-behavioral treatment of borderline personality disorder.* Guilford Press.

Linehan, M. M. (2015). *DBT skills training manual, 2nd ed.* Guilford Press.

Rafaeli, E., Bernstein, D. P., & Young, J. (2011). *Schema therapy: Distinctive features.* Routledge.

Rafaeli, E., Maurer, O., & Thoma, N. C. (2015). Working with modes in schema therapy. In *Working with emotion in cognitive-behavioral therapy: Techniques for clinical practice.* (pp. 263–287). The Guilford Press.

Simons, D. J., & Chabris, C. F. (1999). Gorillas in Our Midst: Sustained Inattentional Blindness for Dynamic Events. *Perception, 28*(9), 1059–1074.

Sockolov, M. (2018). *Practicing Mindfulness: 75 Essential Meditations to Reduce Stress, Improve Mental Health, and Find Peace in the Everyday.* Althea Press.

Stern, D. N. (1985). *The Interpersonal World of the Infant: A View from Psychoanalysis and Developmental Psychology.* Basic Books.

Vygotsky, L. S. (1978). *Mind in Society: The Development of Higher Psychological Processes.* Harvard University Press.

Welch, M. G. (1988). *Holding Time.* Simon & Schuster.

Welch, M. G. (2016). Calming cycle theory: the role of visceral/autonomic learning in early mother and infant/child behaviour and development. *Acta Paediatrica, 105*(11), 1266–1274.

Young, J. E., Klosko, J. S., & Weishaar, M. E. (2003). *Schema therapy: A practitioner's guide.* Guilford Press.
